# Long-term repair of porcine articular cartilage using cryopreservable, clinically compatible human embryonic stem cell-derived chondrocytes

**DOI:** 10.1038/s41536-021-00187-3

**Published:** 2021-11-23

**Authors:** Frank A. Petrigliano, Nancy Q. Liu, Siyoung Lee, Jade Tassey, Arijita Sarkar, Yucheng Lin, Liangliang Li, Yifan Yu, Dawei Geng, Jiankang Zhang, Ruzanna Shkhyan, Jacob Bogdanov, Ben Van Handel, Gabriel B. Ferguson, Youngjoo Lee, Svenja Hinderer, Kuo-Chang Tseng, Aaron Kavanaugh, J. Gage Crump, April D. Pyle, Katja Schenke-Layland, Fabrizio Billi, Liming Wang, Jay Lieberman, Mark Hurtig, Denis Evseenko

**Affiliations:** 1 Department of Orthopaedic Surgery, Keck School of Medicine of USC, University of Southern California (USC), Los Angeles, CA 90033 USA; 2 Department of Orthopaedic Surgery, Nanjing First Hospital, Nanjing Medical University, Nanjing, Jiangsu 210006 China; 3 Department of Orthopaedic Surgery, Zhongda Hospital, School of Medicine, Southeast University, Nanjing, Jiangsu 210009 China; 4 Department of Orthopaedics, Affiliated Jiangning Hospital with Nanjing Medical University, Nanjing, Jiangsu 211100 China; 5 Department of Orthopaedic Surgery, Huai’an First People’s Hospital, Nanjing Medical University, Huai’an, China; 6 Department of Orthopaedic Surgery, Sir Run Run Hospital, Nanjing Medical University, Nanjing, 211166 China; 7 State Key Laboratory of Oral Diseases, National Clinical Research Center for Oral Diseases, Department of Oral and Maxillofacial Surgery, West China Hospital of Stomatology, Sichuan University, Chengdu, 610041 China; 8 The Natural and Medical Sciences Institute (NMI) at the University of Tübingen, Reutlingen, Germany; 9 Department of Stem Cell Research and Regenerative Medicine, USC, Los Angeles, CA 90033 USA; 10 Department of Orthopaedic Surgery, David Geffen School of Medicine, UCLA, Los Angeles, CA 90095 USA; 11 Department of Microbiology, Immunology, and Molecular Genetics, UCLA, Los Angeles, CA USA; 12 Eli and Edythe Broad Center of Regenerative Medicine and Stem Cell Research, UCLA, Los Angeles, CA USA; 13 Molecular Biology Institute, UCLA, Los Angeles, CA USA; 14 Jonsson Comprehensive Cancer Center, UCLA, Los Angeles, CA USA; 15 Department of Biomedical Engineering, Eberhard Karls University Tübingen, Tübingen, Germany; 16 Cluster of Excellence iFIT (EXC 2180) “Image-Guided and Functionally Instructed Tumor Therapies”, Eberhard Karls University Tübingen, Tübingen, Germany; 17 Dept. of Medicine/Cardiology, UCLA, Los Angeles, CA 90095 USA; 18 Ontario Veterinary College, Department of Clinical Studies, University of Guelph, Guelph, ON Canada

**Keywords:** Stem-cell research, Preclinical research, Embryonic stem cells, Regeneration

## Abstract

Osteoarthritis (OA) impacts hundreds of millions of people worldwide, with those affected incurring significant physical and financial burdens. Injuries such as focal defects to the articular surface are a major contributing risk factor for the development of OA. Current cartilage repair strategies are moderately effective at reducing pain but often replace damaged tissue with biomechanically inferior fibrocartilage. Here we describe the development, transcriptomic ontogenetic characterization and quality assessment at the single cell level, as well as the scaled manufacturing of an allogeneic human pluripotent stem cell-derived articular chondrocyte formulation that exhibits long-term functional repair of porcine articular cartilage. These results define a new potential clinical paradigm for articular cartilage repair and mitigation of the associated risk of OA.

## Introduction

Cell therapy has been used successfully in the clinic for more than 50 years in the form of hematopoietic stem cell transplantation^1^. This pioneering work illuminated the need for HLA-matched donors due to graft versus host disease (GVHD) encountered during allogenic transplants, in which donor lymphocytes reacted against host tissues. In the case of allogenic solid organ transplantation, immunosuppression of the host is often required for extended periods^2^. These aforementioned limitations in the availability and compatibility of donor tissue have prompted the search for other solutions which now potentially include human embryonic stem cell- (hESC) and induced pluripotent stem cell-derived (iPSC) cells and tissues^3,4^. The field of PSC-based regenerative medicine has advanced quickly as both iPSC- and ESC-derived cell therapies are in clinical trials^5^, with transplants into immunoprivileged sites such as the eye leading the way.

In the orthopedic field, reparative therapy for articular cartilage defects has classically relied on endogenous cells via the microfracture technique. In this procedure, channels are created through the subchondral plate into the bone marrow to allow bone marrow stromal cells (BMSCs)/skeletal stem cells with chondrogenic potential to enter the defect and generate neocartilage^6^. The reparative fibrocartilage produced following microfracture is often biomechanically inferior to the surrounding hyaline articular cartilage, as MSCs are undergoing chondrogenesis in an inflammatory microenvironment^7^ and in the absence of inductive cues such as BMP-2;^8^ augmented microfracture techniques that address some of these potential limitations are being actively explored^9^. More recently, autologous chondrocyte implantation^10^ (ACI) and variations thereof including MACI^11^ (matrix-associated ACI) that rely on expansion and reimplantation of chondrocytes from the patient, have been adopted. The long-term results of ACI-based procedures appear superior to microfracture, with reduced graft failure and improved patient-reported outcomes^12,13^. Despite overall satisfactory outcomes, many patients still experience complications including graft integration failure, inferior quality of neocartilage (hyaline vs. fibrocartilage), donor site morbidity, osteoarthritis^14^ and chronic pain. Additionally, these approaches require two staged surgical procedures to perform the actual repair, increasing the overall cost and logistic complexity. This has fueled the search for allogeneic sources that may provide more cells with superior chondrogenic capacity without immunocompatibility issues or the need for multiple surgeries including juvenile cartilage^15^ and PSCs.

Generation of articular chondrocytes from PSCs has been challenging as most chondrogenic cells during development are fated to undergo hypertrophy and endochondral ossification rather than adopt an articular chondrocyte identity^16^. We^17,18^ and others^19–22^ have generated articular-like chondrocytes from human pluripotent stem cells; we have subsequently shown that stable articular chondrocytes produced from GFP^+^ PSCs can engraft, integrate into and repair osteochondral defects in small animal models^18^. Moreover, these human cells produce all layers of hyaline cartilage after 4 weeks in vivo, including a PRG4^+^ superficial zone^18^. However, production scaling and assessment of long-term, clinically relevant functionality has so far limited the development of these protocols. The Yucatan minipig presents an excellent model for pre-clinical assessment of potential orthopedic therapies due to structural similarities, comparable thickness of articular cartilage and the ability to create defects of substantial volume;^23–25^ in addition, their size allows for cost-efficient care and observation for extended periods of time. Here we present data demonstrating long-term functional repair of porcine full-thickness articular cartilage defects with hyaline-like cartilage by scalable production of clinical grade hESC-derived immature articular chondrocytes.

## Results

### Scaled production and formulation optimization of hESC-derived chondrocytes

The main purpose of this study was to assess the long-term therapeutic potential of hESC-derived chondrocytes in a porcine model of focal articular cartilage injury. We have previously defined a protocol for the generation of articular cartilage-like chondrocytes from human PSCs^17^. Cells generated using this technique are immature based on their transcriptional signature and expression of immature chondrocyte markers but can mature upon implantation in vivo, evidencing appropriate expression of superficial zone markers and lack of hypertrophy^18^. For this study, we have adapted our previous protocols^17,18^ initially developed for H1 and H9 lines, to utilize the research grade hESC line ESI-017^26^. This specific line was selected because a cGMP version of this line is fully compliant with all current FDA regulations and can be advanced into human clinical trials without any regulatory restrictions.

**Fig. 1:**
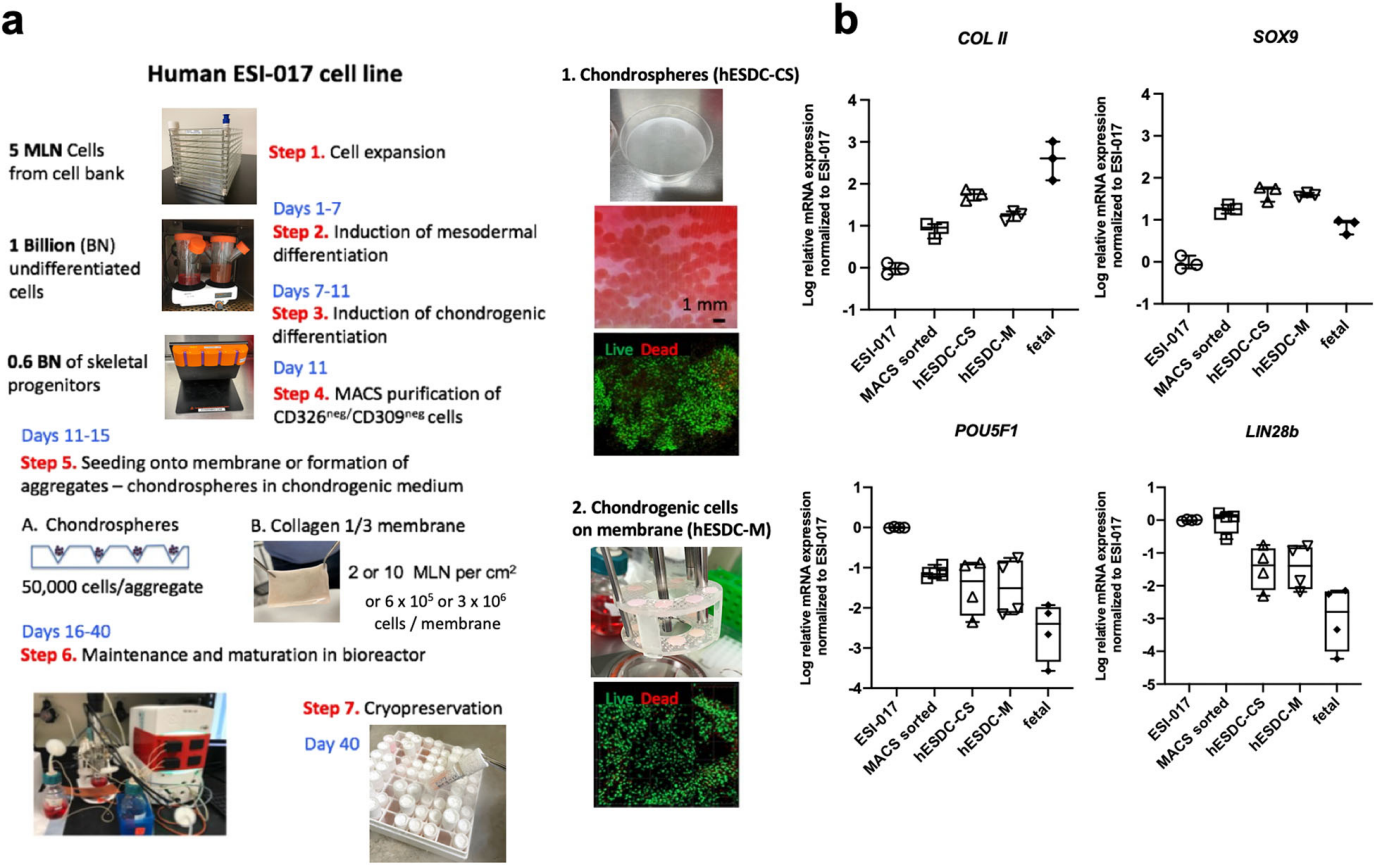
Scale up and formulation of cGMP-grade hES-derived chondrocytes. **a** Schematic depicting the large-scale production of chondrocytes in 2 different formulations from ESI-017 cells. Pre-chondrocytes were seeded onto clinically-used porcine collagen I/III membranes (M) or aggregated to generate chondrospheres (CS). Cells were expanded and then cryopreserved under optimal conditions described in Supplementary Fig. 1. **b** qPCR for chondrogenic and pluripotent genes at different stages of in vitro differentiation or in vivo fetal ontogeny (14–17 weeks). *n* = 3–4 different batches or biological replicates (fetal); data presented as box and whisker plots showing all points.

In order to generate sufficient numbers of clinical grade hESC-derived chondrocytes for cartilage defect repair, hESCs were first expanded in hESC-qualified Matrigel and induced into mesodermal differentiation (d1–7) followed by chondrogenic differentiation (d7–11; Fig. 1a, Supplemental Materials). At d11, mesodermal skeletal progenitors were isolated using MACS to deplete for epithelial (undifferentiated and epidermal, EpCAM/CD326^+^) and cardiovascular mesodermal (KDR/CD309^+^)^27^ cells (Fig. 1a).

During the optimization stage we produced and tested 2 different formulations of hESC-derived chondrocytes: chondrospheres (CS) and chondrocytes integrated onto a collagen I/III membrane previously approved for clinical use^28^ (Cartimaix; Matricel). For chondrosphere production, we used a commercially available low attachment plate with a patterned floor designed to generate chondrospheres of uniform size and quality from d11 MACS-purified chondrogenic cells (Fig. 1a). In parallel, collagen membranes were sized to 6 mm (0.28 cm^2^) with a biopsy punch and seeded with purified skeletal progenitors isolated after MACS and transferred aseptically into the bioreactor. We then used a continuous perfusion bioreactor system (Fig. 1a) for expansion and chondrocyte maturation for an additional 25 days to provide a stable microenvironment with precise control of gases, nutrients and physical parameters such as shear stress. We confirmed that ESI-017 cells responded to our established protocol by upregulating chondrogenic and downregulating pluripotency genes during the course of the manufacturing process (Fig. 1b).

We then tested cryopreservation media (Supplementary Fig. 1) for each of these formulations to support the development of a universal, off-the-shelf potential therapy for articular cartilage defects. After optimization of cryopreservation, both chondrospheres and membranes were revived with using Mesencult^TM^ ACF (Supplementary Fig. 1). These batches routinely yielded dozens of chondrospheres containing ~5 × 10^4^ cells, or low and high dose membranes containing 6 × 10^5^ or 3 × 10^6^ immature chondrocytes each, respectively.

We then compared the ability of two doses of each formulation to support short-term repair of a focal defect in pig articular cartilage (Supplementary Fig. 2). Six millimeter full-thickness cartilage defects were created to the depth of the subchondral plate in the femoral condyle and low and high doses of hESC-derived chondrospheres or chondrocytes on membranes were implanted (Supplementary Fig. 2a); empty membranes or glue alone were used as controls. One month later, injured areas were assessed for evidence of tissue integration, matrix production, fibrosis and presence of Ku80+ human cells;^29^ detailed biomechanical mapping was also conducted to determine biomechanical characteristics of the healing defects. These results (Supplementary Figs. 2 and 3) clearly demonstrated that a high dose of chondrocytes embedded in collagen membranes was better maintained in the injury site and supported superior functional repair without eliciting a heightened immune response (Supplementary Fig. 4^30^). Based on these data, we proceeded with membrane-bound hESC-derived chondrocytes (hESDC-M) for further characterization and functional testing.

### Characterization and developmental status of hESDC-M

**Fig. 2:**
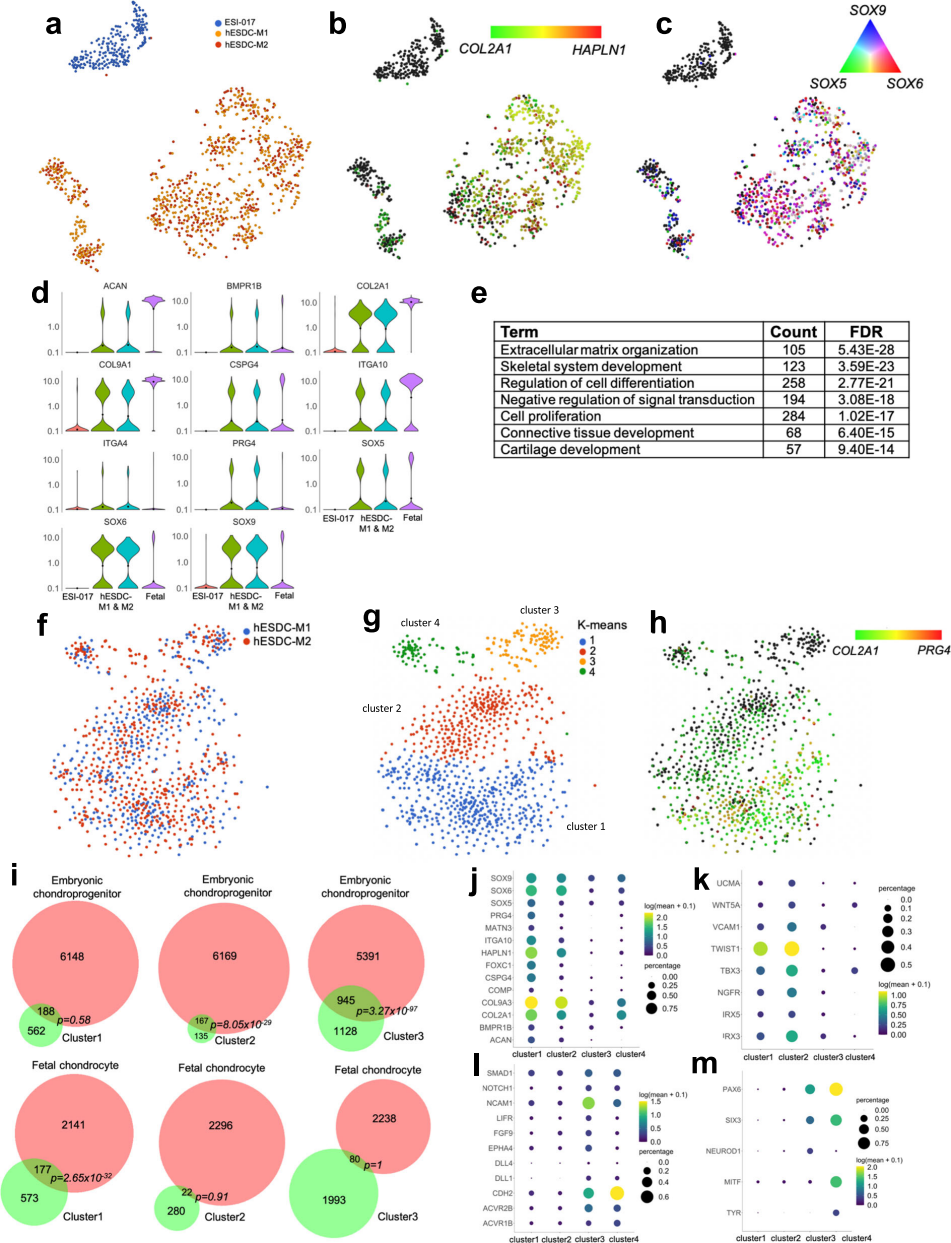
Transcriptional profiling of membrane embedded hESDC-M. **a** t-SNE plot of 1173 single cells sequenced, generated from ESI-017 cells (*n* = 1, blue) or hES-derived chondrocytes digested from membranes at d40 of differentiation (*n* = 2 batches in red and orange). **b, c** t-SNE plots depicting expression of indicated genes at single cell resolution. **d** Violin plots for gene expression of selected chondrogenic genes; fetal chondrocyte expression data are shown for reference. **e** Selected gene ontology (GO) categories enriched in hESDC-M vs. ESI-017 cells based on genes with FDR < 0.05, >2-fold change. **f** Re-clustering, **g** k-means clustering and **h** *PRG4* and *COL2A1* expression levels of 965 hESDC-M cells. **i** Venn diagrams demonstrating overlap of genes strongly enriched (biomarker genes) in the indicated cluster with (top) genes enriched in embryonic chondroprogenitors isolated from 5–6 wk limbs vs. fetal chondrocytes isolated at 17 wks from knee joints analyzed by scRNA-Seq or (bottom) vice versa. **i**–**m** Expression of selected and biomarker genes in each cluster of hESDC-M.

We have previously shown chondrocytes derived from hESCs using our protocol may lie between fetal and adult cells on the developmental timeline^18^. These experiments were previously conducted on cells isolated from chondrospheres using bulk RNA-seq. To better understand the heterogeneity present in hESDC-M, we performed single cell RNA-seq (scRNA-seq) on cells isolated from two different batch production runs and compared them to undifferentiated ESI-017 cells (Fig. 2). Importantly, few to none of the cells demonstrated expression of pluripotency genes (*POU5F1, LIN28A or ZFP42*; Supplementary Fig. 5a), suggesting limited potential for generation of teratomas upon transplantation in vivo. Both production runs contained similar cell types as shown by objective clustering (Fig. 2a), with the absolute majority of cells present being positive for chondrogenic markers including *COL2A1, HAPLN1*, and *SOX5/6/9*, (Fig. 2b–d). This was affirmed by gene ontology analysis of genes enriched in the hESDC-M (FDR < 0.05, >2-fold change) as demonstrated by significant over-representation of genes related to cartilage development, matrix production and lineage commitment (Fig. 2e). Objective clustering of hESDC-M defined 4 subtypes of cells present on the membranes, potentially representing a continuum of chondroinduction and chondrogenesis and/or chondrogenic cells with different mesodermal origins;^31^ clusters 1 and 2 were the most enriched for *COL2A1* and *PRG4* (Fig. 2f–h). To define how these subtypes of hESDC-Ms compare with different stages of human ontogeny and human bone marrow stromal cells cultured on membranes (hBMSC-M), we performed scRNA-seq at multiple stages of human chondrogenic ontogeny and on hBMSC-M (Supplementary Figs. 5 and 6). These data defined cluster 1 as the most mature, with highly significant overlap with genes enriched in fetal chondrocytes vs. embryonic chondroprogenitors, including genes encoding ECM proteins such as *COL2A1, PRG4* and *ACAN* (Fig. 2i, j; Supplementary Fig. 6). Clusters 2 and 3 were more closely related to chondroprogenitors and chondroinductive cells present early during development, showing enrichment for genes involved in the generation of chondrogenic condensations and primitive mesoderm including *TWIST1*^32^*, NCAM1* and *CDH2*^33^ (Fig. 2k, l; Supplementary Fig. 6). These data were confirmed by comparison to our previous bulk sequencing data generated at these stages^17,18^ (Supplementary Fig. 6). Cluster 4 contained cells expressing *COL2A1* and neural markers including *PAX6* and *MITF* (Fig. 2m), similar to a population recently described to be present during hIPSC chondrogenesis^34^. We also conducted a trajectory analysis of COL2^+^ cells from each stage of human ontogeny and in vitro differentiation; these results placed hESDC-Ms between the embryonic and juvenile stages of human ontogeny (Supplementary Fig. 6b). Conversely, hBMSC-M cultured under identical conditions to hESDC-M expressed genes associated with terminal chondrogenesis including *COL10A1* and *SPP1* (Supplementary Fig. 5c, d). These data reveal that although the cells present on the membrane are heterogenous, the production process is reproducible and generates mostly immature chondrogenic and chondroinductive cells.

### hESDC-M support long-term repair of articular cartilage in pigs

**Fig. 3:**
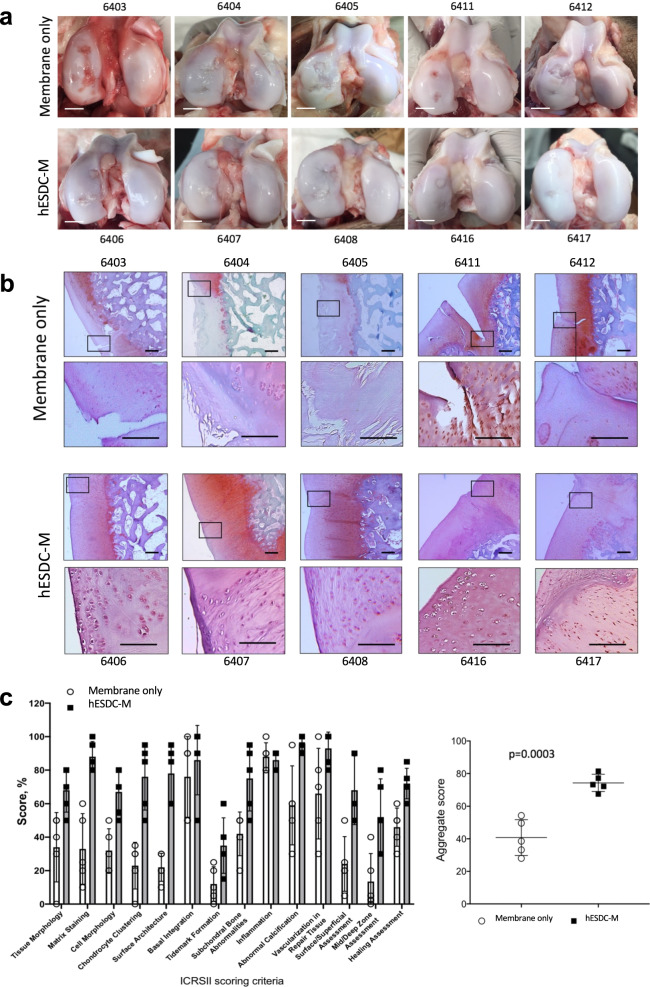
Focal articular cartilage defects treated with hESDC-M show improved repair at 6 months. **a** Gross visual appearance of all 10 defects created in the femoral condyle of control (membrane alone, top row) or treated (hESDC-M, bottom row) Yucatan minipig knees after 6 months. Scale bar = 10 mm. **b** Safranin O/Fast Green staining of the interface between the graft and endogenous tissue or the defect itself (boxes); where the boxed regions are shown at higher magnification below. Scale bar = 100 μm. **c** Histological scoring of sections from control and treated femoral condyles for the 14 parameters comprising the ICRS II cartilage repair scoring system (left); each point represents the average of both defects per animal. (Right) Aggregate score of all 14 parameters over the 10 defects scored. Identifiers above or under images represent each animal. *p*-value was calculated using unpaired Student’s t-test; data presented as mean ± SD.

In order to assess the therapeutic potential of hESDC-M, we designed a long-term clinically relevant experiment in which either membranes alone or membranes with cells were implanted into pig articular cartilage defects and assessed 6 months later. Pigs in each group (*n* = 5) had 2, 6 mm full-thickness cartilage defects created with an average of 7 mm apart in their femoral condyles within the load bearing areas and were treated with either membranes alone or hESDC-M; two animals were used as sham controls with no cartilage defects generated. Cell implants were thawed and washed in fresh X-Vivo media approximately 1 h prior to implantation, applied to the defects, and were fixed in place with fibrin glue. After 6 months, pigs were euthanized and cartilage assayed using morphological, histological and biomechanical methods. Visually, defects from all pigs transplanted with cells uniformly evidenced substantially less degeneration in and around the injury site (Fig. 3a). Sham operated animals showed no noticeable morphological or biomechanical differences with non-operated knees (data not shown). At the microscopic level, defects treated with cells contained neocartilage with more proteoglycan deposition and better integration of the new cartilage tissue with the non-injured surrounding matrix (Fig. 3b, Supplementary Fig. 4e). To quantify the extent of regeneration provided by cells, all 10 defects per group were scored by 2 blinded observers using the International Cartilage Repair Society (ICRS) II histological assessment system^35^ (Fig. 3c). This scoring system grades 14 criteria relevant to cartilage repair and provides a comprehensive view of the utility of potential treatment. Scores from each observer for each defect were averaged to provide a composite for each criterion. These data showed significantly better outcomes for defects treated with hESDC-M (Fig. 3c). This was confirmed by synovitis scoring and staining for inflammatory infiltrates, which showed no difference between animals implanted with empty membranes vs. those receiving hESDC-M, further supporting the low immunogenicity of hESDC-M (Supplementary Fig. 4). Moreover, morphological signs of synovitis were more prominent in pigs treated with membranes only, likely reflecting progression of degenerative joint disease in this group; however, this difference did not reach statistically significant values (Supplementary Fig. 4d). Histological analysis of the subchondral bone showed no major differences between the groups (data not shown).

**Fig. 4:**
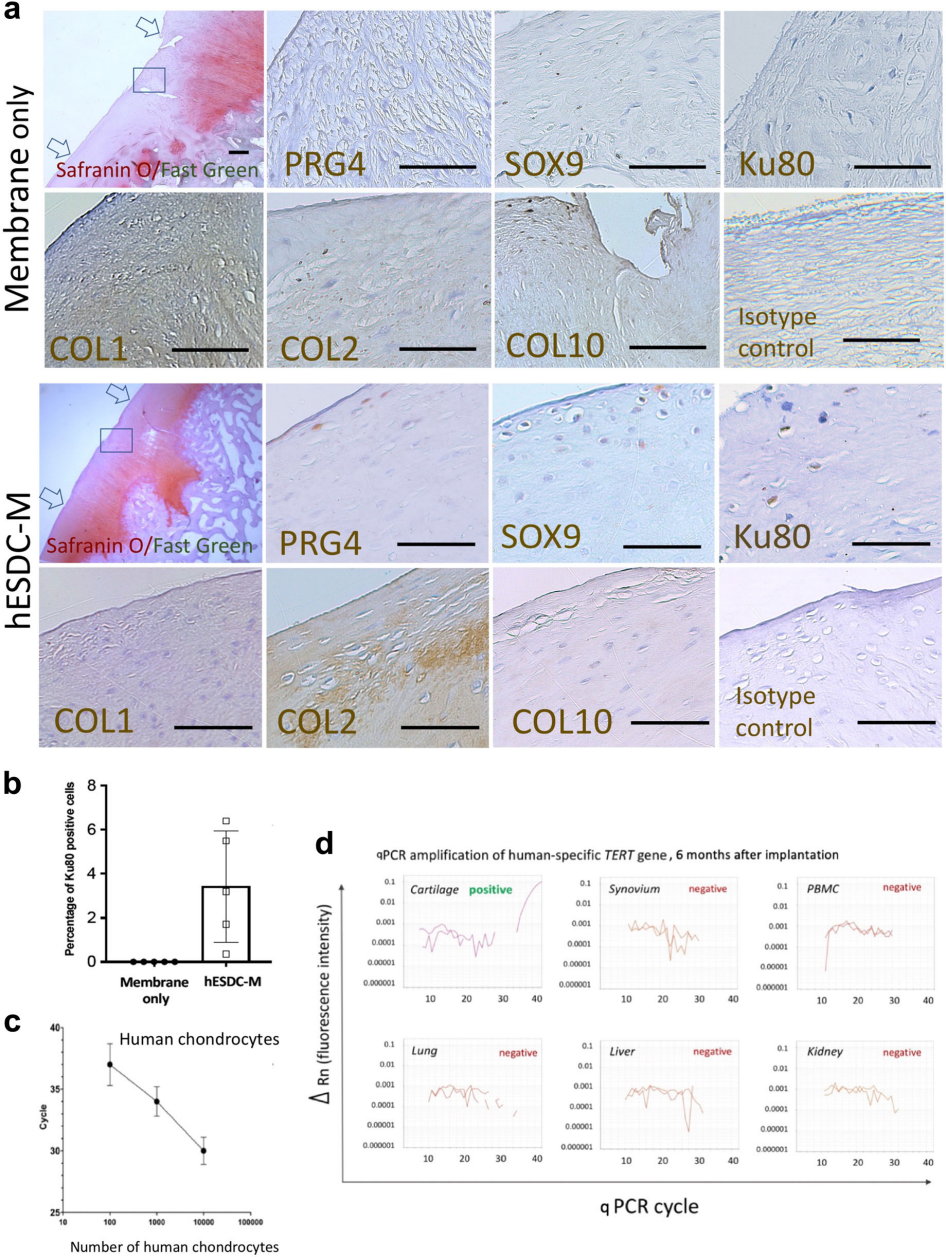
hESDC-M treated defects evidence superior repair and contain both human and pig cells at 6 months. **a** Histochemical staining of the full defect (indicated by arrows) for Safranin O/Fast Green to assess glycosaminoglycans for control (membrane only) and treated (hESDC-M) animals. Representative images of immunohistochemical staining of the boxed area for human-specific antigen Ku80 and zonal markers of articular cartilage for both control and treated femoral condyles are shown and highlighted with black triangles; scale bar = 200 μm. **b** Quantification of Ku80 + cells (mean ± SD of 5 biological replicates). **c** qPCR analysis of human *TERT* gene. Standard curve constructed with human chondrocyte genomic DNA allowed reliable detection of as few as 100 human cells (mean ± SD of 3 biological replicates). **d** Genomic DNA extracted from the indicated tissues was analyzed for the human *TERT* gene. Representative amplification plots are shown; human cells were detected in all defects of animals treated with hESDC-M. PBMCs = peripheral blood mononuclear cells.

At the molecular level, cartilage in the defects treated with cells more closely resembled the surrounding tissue. In membrane only defects, much of the new tissue was inferior as evidenced by collagen 1 and collagen X staining coupled with substantially reduced proteoglycan content (Fig. 4a). In contrast, defects transplanted with hESDC-M evidenced appropriate stratification of the neocartilage as demonstrated by superficial production of lubricin (PRG4) and localization of SOX9^+^ cells (Fig. 4a). Moreover, substantial production of collagen II in the transitional zone was primarily observed in defects treated with cells, showing similar collagen deposition compared to normal pig cartilage^36^ (Supplementary Fig. 9). Notably, even after 6 months following implantation, small clusters of Ku80^+^ human cells^29^ were identifiable in all treated animals (Fig. 4b). With the safety profile of hES-derived chondrocytes in mind, we explored the biodistribution of human cells by using a sensitive PCR-based assay to detect human telomerase (*TERT*) in cartilage, synovium, peripheral blood and major organs (Fig. 4c, d). While the presence of human cells in the repaired articular cartilage was confirmed and represented roughly 4% of total cells (Fig. 4b), levels of human DNA in all other tissues analyzed, including synovium, was below the threshold of detection, indicating that transplanted cells do not leave the defect following implantation after 6 months.

**Fig. 5:**
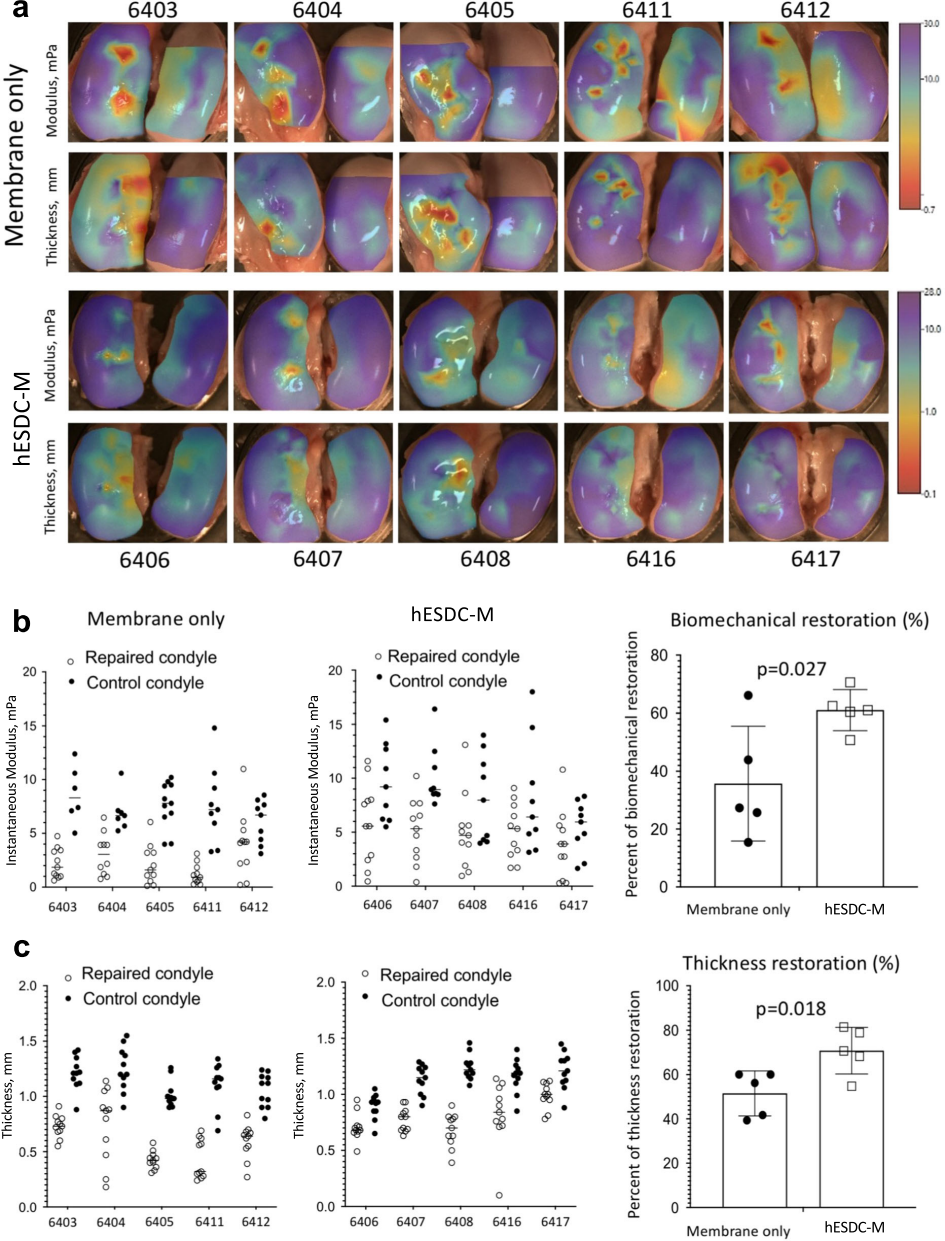
hESDC-M elicit biomechanically superior articular cartilage repair long term in porcine knees at 6 months. **a** Heat maps depicting scanning indentation and thickness of femoral condyles generated using Mach-1 bioindentor; scale bars for instantaneous modulus (top rows) and thickness (bottom rows) are shown on the right. **b** Data points for instantaneous modulus or **c** thickness measurements for each defect (2 per condyle) were merged into one aggregate measure and compared to the same area of the uninjured condyle of the same pig; values for both defects per animal were averaged and the plotted as controls (membrane only) vs. treated (hESDC-M) as a function of average uninjured condyle measurements to calculate percent restoration (right). Identifiers represent individual animals (*n* = 5). *p*-values were calculated using unpaired Student’s *t*-test; data presented as mean ± SD.

As a measure of functional repair, we evaluated the biomechanical properties of the neocartilage in control and cell-treated defects (Fig. 5). The surfaces of both femoral condyles were biomechanically mapped in all 10 pigs to assess cartilage stiffness and thickness (Fig. 5a). The instantaneous modulus, a measure of compressibility of cartilage, was higher in most defects treated with cells and more similar to native cartilage (Fig. 5b). As expected from the histology, cartilage in and around the defects treated with cells was thicker than in defects treated with membranes alone (Fig. 5a, c). Finally, the biomechanical properties of all repaired defects in each group were merged into a composite score; although animals treated with cells did not achieve full restoration of biomechanics, they were significantly improved as compared to defects treated with membranes alone (Fig. 5b, c). These results indicate that hESDC-M promote the formation of hyaline cartilage that is biomechanically similar to native tissue for at least 6 months after implantation.

### hESDC-M secrete chondroinductive factors that induce chondrogenesis from porcine BMSCs

**Fig. 6:**
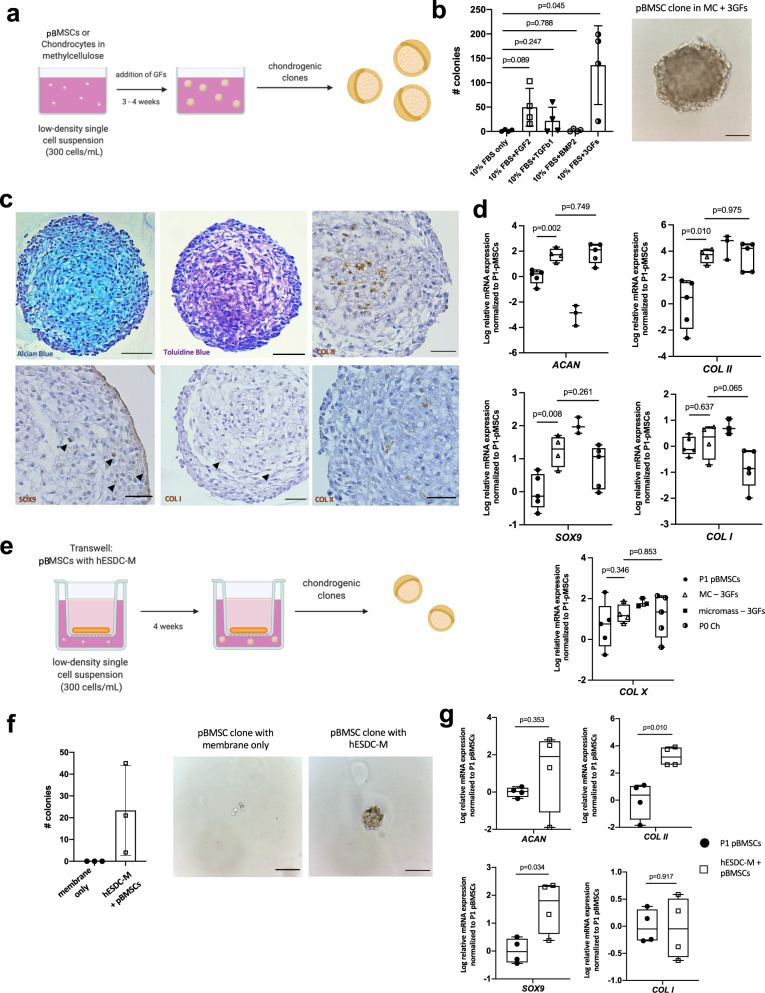
hESDC-M produce paracrine factors that drive chondrogenesis of endogenous cells. **a** Schematic depicting the methylcellulose (MC) culture method created with Biorender.com. **b** Clonogenicity of porcine bone-marrow derived stromal cells (pBMSCs) in MC with different GFs; *n* = 4 biological replicates. Representative image of a pBMSC-derived colony after 4 weeks in MC with 3 growth factors (right); scale bar = 100 μm. **c** Alcian Blue and Toluidine Blue staining (left, middle) and immunohistochemical staining various chondrogenic markers of pBMSCs grown in MC with 3 GFs after 4 weeks. Scale bar = 100 μm. **d** qPCR of chondrogenic genes (*n* = 5 biological replicates for P1 pBMSCs and P0 Ch, n = 4 for pBMSCs in MC, and *n* = 3 for pBMSCs cultured micromass). **e** Schematic of the MC with Transwell culture method. **f** Clonogenicity of pBMSCs in MC with a membrane only or hESDC-M in Transwell after 4 weeks, *n* = 3 biological replicates per group. Representative images of pBMSCs in the Transwell after 4 weeks are shown; scale bar = 100 μm. **g** qPCR of chondrogenic genes from pBMSCs grown in Transwell with hESDC-M, *n* = 4 biological replicates. *p*-values were calculated with an unpaired Student’s t-test; data presented as mean ± SD or box and whisker plots showing all points.

Given that the majority of hyaline-like neocartilage was contributed by pig cells, we assessed the chondroinductive, paracrine effects of hESDC-M on pig BMSCs (Fig. 6) and chondrocytes (Supplementary Fig. 8), the cells likely responsible for generating neocartilage in the pig model. We utilized a methylcellulose (MC)-based culture method to assess both clonality at a single cell level and capacity of pig BMSCs to undergo chondrogenesis in vitro (Fig. 6a). Growth factors of the TGF-β^37^, FGF^38^ and BMP^39^ families are known to be chondroinductive during development and following injury. Upon this basis, we selected three growth factors (FGF-2, BMP-2, and TGF-β1, 3GFs) produced by hESDC-M (Supplementary Fig. 7) and added them to the MC-based media, showing that a combination of all 3 growth factors yielded the most clones (Fig. 6b). Notably, BMP-2 was not secreted by hBMSCs (Supplementary Fig. 7d), suggesting that endogenously activated BMSCs may not produce sufficient chondroinductive factors to generate hyaline-like neocartilage as was observed with hESDC-M. These colonies produced proteoglycans and other chondrogenic markers comparable to native articular chondrocytes cultured in the same method (Fig. 6c, Supplementary Fig. 8e). Chondrogenic gene expression similar to that of native pig articular chondrocytes was evident when comparing BMSCs cultured in MC with 3GFs, with substantial induction of chondrogenic genes versus starting BMSCs (Fig. 6d). Moreover, culture in MC + 3GFs yielded chondrogenic gene expression similar to micromass culture of pig BMSCs + 3GFs, the standard method for generating chondrocytes from BMSCs in vitro. To assess whether paracrine factors produced by hESDC-M could promote chondrogenesis from pig BMSCs, we employed a co-culture system with Transwell inserts and MC (Fig. 6e). After 4 weeks of co-culture, clonality and chondrogenesis showed the same trends as BMSCs cultured with all 3GFs (Fig. 6f, g); expression of collagen X was undetectable. Importantly, hESDC-M also supported clonal chondrogenesis from pig chondrocytes (Supplementary Fig. 8), suggesting two possible cellular sources for neocartilage following hESDC-M implantation. These data indicate secreted proteins such as BMP-2 produced by hESDC-M can promote induction of articular-like chondrogenesis from BMSCs, implying that the paracrine factors supplied are crucial to the generation of functionally superior neocartilage.

## Discussion

We have shown that hESC-derived chondrocytes administered as a cryopreservable, membrane-embedded formulation support clinically relevant, long-term repair of full-thickness articular cartilage defects in pigs. At the molecular level, prior to implantation, membranes were found to contain no detectable residual hESCs and be populated with immature chondrocytes resembling both embryonic chondroprogenitors and juvenile articular chondrocytes. Based on the high expression levels of *SOX5/6/9* and genes associated with proliferation, it is likely that these immature articular chondrocytes mature in vivo to upregulate matrix production and assume a proper zonal identity^18^. Critically, the hyaline-like cartilage found in repaired defects had similar biomechanical properties to naïve tissue, further supporting the concept that hESDC-M can adopt adult-like properties upon transplantation and integration with surrounding cells and/or support recruitment and differentiation of chondrogenic cells into hyaline cartilage via paracrine factors.

Despite being a xenograft in immunocompetent recipients with no immunosuppression, we found no evidence of local inflammation or immune cell infiltration. In light of the clinical use of particulate juvenile chondrocytes and osteochondral transplants as allograft material^40,41^, this is not entirely unexpected. However, other studies have demonstrated that xenografted articular chondrocytes in the knee can elicit a severe immune response^42^. Once transitioned to human trials of allogenic hESDC-M for focal articular cartilage repair, patients will have to be evaluated carefully to determine which of them may require additional screening or exclusion based upon the degree of joint inflammation or other systemic diseases. Additionally, although we did not detect any peripheral dissemination of human cells or residual pluripotent cells in the current work using highly sensitive analyses, each of these will require additional testing to validate the safety profile of this potential cell therapy and the release criteria for each production batch.

The significant biomechanical improvement seen in defects treated with human cells is very likely the result of both autocrine and paracrine mechanisms. This suggests that factors secreted by hESDC-M such as BMP-2 can recruit endogenous cells capable of producing hyaline-like cartilage and support their differentiation down this path, whereas recruited hBMSCs do not secrete the factors necessary for generating healthy cartilage. Murphy et al. recently demonstrated that implantation of hydrogels loaded with BMP-2 and sVEGFR-1 could significantly improve the outcome of microfracture in both young and old mice^9^. Moreover, they show that treatment with BMP-2 alone was insufficient to generate articular cartilage following microfracture, clearly indicating that induction of an articular-like fate from BMSCs requires more than one input. Consistent with other studies^43,44^ our data show that BMP-2 was not secreted at detectable levels by hBMSCs but was robustly generated by hESDC-M; moreover, the scRNA-Seq data presented here clearly identify a population similar to primitive, chondroinductive cells present in the developing limb. The potential chondrogenic paracrine factors secreted by hESDC-M may modulate the microenvironment of the defect to promote migration and differentiation of endogenous cells into articular cartilage. It will be important to assess the response when hESDC-M are used in injuries that occurred significantly before transplantation, as acute inflammation will have subsided and chronic inflammation may be elevated.

There are caveats to the data presented here. Although pig models of cartilage repair are commonly used^23,24^, some biomechanical differences between human and pig joints are well documented^25^. Porcine knees have significantly softer cartilage and longer trochlea than those of humans, putting the joint in a constant state of flexion. Moreover, humans are more active than pigs, leading to higher load bearing properties on average. These parameters could influence implanted cells in ways different than documented here. In addition, focal articular cartilage defects in humans often involve the cartilage without reaching the subchondral space^24^ and are often latent for some time prior to a repair procedure while in the current study, focal full-thickness lesions were freshly made; in addition, we did not address the impact on subchondral bone via microCT although general histological analysis of the subchondral bone was conducted. Many model species including large animals can spontaneously heal full-thickness defects at adolescent ages^23,24^, which may highlight an important association with age and lower inflammation in the joint after experimental assessment^23^, further underscoring the lack of a perfect pre-clinical model for articular cartilage repair. In line with this, integration of graft tissue with endogenous articular cartilage is a major concern for cell-based therapies of full-thickness defects^14,23^ as transplanted autologous chondrocytes do not appear to consistently attract cells with capacity to promote bridging; graft integration failure has been documented in a pig model of marrow stimulation as well^23^. We did not include control groups in the current study that tested transplantation of autologous chondrocytes, BMSCs or allogeneic hBMSCs as these cells have been vigorously evaluated in past studies^23,45,46^ and have not demonstrated long-term retention of implanted cells. Based on the relatively primitive nature of hES-derived chondrocytes, we speculate their increased motility, secretome and integration will support better graft integration outcomes in full-thickness defects. Finally, pain and the associated loss of mobility are of significant concern in patients with focal cartilage lesions^14^. Although we did not directly assess joint usage or gait as a surrogate for pain in this study, future clinical evaluations of hESDC-M at all stages will include assessments of pain with reproducible test re-test results^47^.

In spite of these potential weaknesses, the presented robust potential of hESDC-M to enact articular cartilage repair as a universal, off-the-shelf product should be explored clinically. Current cell therapies for focal defect repair rely on expansion and implantation of autologous cells (e.g. MACI) or allogenic juvenile articular cartilage which has limited availability. If hESDC-M can enact meaningful repair of focal defects in patients, this represents a unique opportunity to scale an allogenic therapy, providing the possibility of superior functional repair from an inexhaustible source of chondrogenic cells.

## Methods

### General methods

For all experiments, independent experiments with biological replicates were employed to generate data. For in vitro experiments expected to yield large differences, standard practice of using 3 replicates was followed. All statistical methods are described in the figure legends.

### Culture of pluripotent stem cells and generation of skeletal progenitors

For all experiments, research grade ESI-017 were used; the stock line was purchased from BioTime. Cells were cultured on hESC-qualified, LDEV-free Matrigel (Corning # 354277) in mTesR1/Plus media and passaged using ReLeSR (Stemcell Technologies) according to manufacturer’s instructions. Batch scale production of ESI-017 cells was carried out by seeding ~5 million cells in CellSTACK-5 flasks, yielding ~1 billion cells per batch. Each batch was tested for mycoplasma contamination by PCR (Sigma). Cells were then transferred to suspension culture in mTesR1/Plus for up to 5 days in spinner flasks (60 RPM) for additional expansion and differentiation. Differentiation was conducted as described previously^17,18,27^. Briefly, mTesR1/Plus was replaced with Mesoderm Induction Media A (MIM-A) for 3 days [X-Vivo (Lonza) containing ROCK inhibitor (10 μM; Y27623, Tocris), FGF2 (10 ng/ml), Wnt3a (10 ng/ml) and Activin A (10 ng/ml)] followed by MIM-B for 3 days [X-Vivo containing Wnt3a (10 ng/ml), Noggin (50 ng/ml) and FGF-2 (10 ng/ml)]. MIM-B contained no ROCK inhibitor or Activin A to avoid excessive endodermal differentiation. After 7 days of differentiation, when skeletal progenitors were already specified, media was changed to Chondrogenic Induction Media A (CIM-A; X-Vivo containing BMP-4 (10 ng/ml) and FGF-2 (10 ng/ml)) for 4 additional days. For 2 h before starting MACS isolation, ROCK inhibitor was added again to the cell culture media to increase cell survival after enzymatic dissociation and sorting, and these skeletal progenitors were then isolated using MACS by depleting for CD326 (PerCP) and CD309 (PE), yielding ~0.6 billion cells; or 15–20% of total cells. The remaining cells represented undifferentiated cells, endodermal precursors, and also mesodermal cells committed to cardiovascular and hematoendothelial lineages^27^. The purity of cells isolated from MACS was routinely assessed for negativity of CD326 (PerCP) and CD309 (PE) using flow cytometry.

### Generation of chondrospheres (CS)

Skeletal progenitors from MACS were cultured in EZSphere plates (Nacalai USA) at 100,000 cells per well in CIM-B^17,18^ (X-Vivo containing Shh (25 ng/ml), ROCK inhibitor (10 μM), BMP-4 (50 ng/ml), FGF-2 (10 ng/ml), IGF-1 (10 ng/ml) and Primocin (broad spectrum antibiotic, 100 μg/ml)) in 5% oxygen for 3–5 days to form chondrogenic aggregates. After firm aggregates were verified with microscopy, they were transferred to a perfusion bioreactor (Applikon Biotech) in Maturation Media (MM; X-Vivo media containing FGF-2 (10 ng/ml), BMP-4 (1 ng/ml), IGF-1 (10 ng/ml), LIF (50 ng/ml), TGF-β1 (10 ng/ml) and Primocin (100 μg/ml)) until d40 of differentiation. Conditions in the bioreactor were kept at 37 °C, 5% O_2_, 5% CO_2_ and 30 RPM. Fresh media was added at a rate of 1 mL/hour. Upon maturation, chondrospheres contained 5 × 10^4^ cells on average. At d40, chondrospheres were cryopreserved in Mesencult-ACF plus ROCK inhibitor (10 μM) and stored in liquid nitrogen until use.

### Generation of hES-derived chondrocytes on membranes (hESDC-M)

Skeletal progenitors isolated via MACS were seeded onto porcine collagen I/III membranes (Cartimaix; Matricel) sized with a 6 mm biopsy punch at two different amounts (1 or 4 million viable cells), yielding ~6 × 10^5^ or 3 × 10^6^ cells attached to the membranes, respectively. Membranes were then cultured in 5% oxygen for 3–5 days in CIM-B and then transferred to the bioreactor as above for chondrospheres with the exception that rotation was not started until 3 days after transfer of membranes to allow the cells to attach to the membranes. At d40, membranes were cryopreserved using Mesencult-ACF as above for chondrospheres.

### Generation of hBMSCs on membranes (hBMSC-M)

Human bone marrow stromal cells (*n* = 3 donors aged 19, 68, and 87 years (all P1); pooled) were seeded onto porcine collagen I/III membranes (Cartimaix; Matricel) sized with a 6 mm biopsy punch at a total cell number of 3 × 10^6^. Membranes were then cultured in 5% oxygen for 3–5 days in CIM-B, then maintained with MM until d40. At d40, membranes were digested for single cells and scRNA-sequencing was performed.

### Optimization of cryopreservation media

Live cell numbers per batch of chondrospheres or membranes were determined prior to freezing using the Live/Dead Cell Viability Assay (Biovision). Chondrospheres or membranes were then cryopreserved following the manufacturer’s instructions for each product (Prime XV FreezIS, Irvine Scientific; Mesencult-ACF, CryoStor CS5 or 10, mFreSR; Stemcell Technologies). Viability post-thawing from the same batch was compared to the starting viability before freezing.

### Quantitative real-time PCR

Power SYBR Green (Applied Biosystems) RT-PCR amplification and detection was performed using an Applied Biosystems Step One Plus Real-Time PCR machine. The comparative Ct method for relative quantification (2-ΔΔCt) was used to quantitate gene expression, and displayed as Log_10_ of relative expression. TBP (TATA-box binding protein) or RPL7 (ribosomal protein L7) was used for gene normalization. For quantification of human cell numbers in pig samples, a human *TERT* Taqman assay was used (Thermo). A standard curve was created with known numbers of human cells, which both determined the detection threshold as well as allowed calculation of human cell numbers in a sample based on Ct values.

### Primer sequence List

Please see Table 1 for a list of qPCR primer sequences used.

**Table 1:** Primer sequence list.

Gene name	Primer sequence	GenBank accession
hRPL7	Forward: 5′ CCAAATTGGCGTTTGTCATCAG 3′	NM_000971
	Reverse: 5′ GCATGTTAATCGAAGCCTTGTTG 3′	
hTBP	Forward: 5′ TGCACAGGAGCCAAGAGTGAA 3′	NM_001172085
	Reverse: 5′ CACATCACAGCTCCCCACCA 3′	
hCOL2A1	Forward: 5′ TGGACGATCAGGCGAAACC 3′	NM_001844
	Reverse: 5′ GCTGCGGATGCTCTCAATCT 3′	
hSOX9	Forward: 5′ AGCGAACGCACATCAAGAC 3′	NM_000346
	Reverse: 5′ GCTGTAGTGTGGGAGGTTGAA 3′	
hLIN28b	Forward: 5′ CATCTCCATGATAAACCGAGAGG 3′	NM_001004317
	Reverse: 5′ GTTACCCGTATTGACTCAAGGC 3′	
hPOU5F1	Forward: 5′ AGTGAGAGGCAACCTGGAGA 3′	LC006945.1
	Reverse: 5′ CACTCGGACCACATCCTTCT 3′	
pRPL7	Forward: 5′ CAGGATCAGAGGTATCAA 3′	NM_001113217.1
	Reverse: 5′ TATATGGTTCCACAATTCTC 3′	
pACAN	Forward: 5′ CTACGACGCCATCTGCTACA 3′	NM_001164652.1
	Reverse: 5′ CTTCACCCTCGGTGATGTTT 3′	
pCOL2A1	Forward: 5′ GAGAGGTCTTCCTGGCAAAG 3′	XM_021092611.1
	Reverse: 5′ AAGTCCCTGGAAGCCAGAT 3′	
pSOX9	Forward: 5′ CCACCGAAGAAAGACCGTAA 3′	NM_213843.2
	Reverse: 5′ CTTGGAATGTGGGTTCGAGT 3′	
pCOL1A1	Forward: 5′ CCAGTCACCTGCGTACAGAA 3′	LC223106.1
	Reverse: 5′ ACGTCATCGCACAACACATT 3′	
pCOLXA1	Forward: 5′ ACTTCTCCTACCACATTC 3′	NM_001005153.1
	Reverse: 5′ CCATACCTGGTCATTATCT 3′	

h = human, p = pig.

### Large animal model of articular cartilage repair

Yucatan minipigs were purchased from S & S farms at 6 months of age and housed under the supervision of the USC Department of Animal Resources (DAR). All pre-operative, surgical and post-operative procedures were conducted following USC DAR guidelines and were overseen by the USC Institutional Animal Care and Use Committee (IACUC). Five animals per group (main study) with 2 defects each were included based on power calculations to yield the minimum number of animals projected to acquire statistically significant results. Briefly, we used the formula $n = 1 + 2C\left( {\frac{s}{d}} \right)^2$ based on a 30–40% difference and 20% standard deviation between experimental groups, with these parameters based on our previous studies of cell-based focal repair of articular cartilage in large animal models^49–52^. Animals were anesthetized for surgery using Telazol/Xylazine 2.2–4.4 mg/kg administered intramuscularly. Medial para-patellar arthrotomy was performed by inserting microsurgical scalpel medially and proximally to the insertion of the patellar tendon on the tibia and extending it proximally until the attachment of the quadriceps muscle. The medial margin of the quadriceps was separated from the muscles of the medial compartment. The joint was extended and the patella dislocated laterally. The joint was then fully flexed to expose to expose the patellar groove. A 6 mm-diameter disposable biopsy punch was used to create two full-thickness injuries in the articular cartilage, without perturbation of the underlying subchondral bone. The wound bed was cleaned with sterile cotton applicators. One hundred *μ*L of fibrin glue (Ethicon) was used to suspend the chondrospheres in a sterile formed plug for implantation. The formed chondrosphere plug was then transferred and compressed into the defect. Both the chondrospheres and hES-derived chondrocyte membranes were sealed with several droplets of fibrin glue to allow setting of the implantation. No randomization was applied. For animals receiving chondrospheres in the pilot study, surgical fibrin glue with or without chondrospheres was applied directly to the defect areas. For animals receiving membranes, pre-sized membranes were applied to the defect and secured with fibrin glue. Arthrotomies were then relieved, wounds sutured and animals monitored for recovery. Following 1 or 6 months, animals were euthanized after receiving anesthesia as above using pentobarbitol (100 mg/kg).

### Tissue collection and digestion

Adult human primary tissue samples were obtained from National Disease Research Interchange (NDRI). Fetal and embryonic tissue samples were obtained from Novogenix Laboratories. All donated material was anonymous, carried no personal identifiers and was obtained after informed consent. Sex of the specimens was unknown. Human primary tissues, hES-derived chondrocytes on membranes, or hBMSCs on membranes were manually cut into small pieces and digested 4–16 h at 37 °C with mild agitation in digestion media consisting of DMEM/F12 (Corning) with 10% FBS (Corning), 1 mg/mL dispase (Gibco), 1 mg/mL type 2 collagenase (Worthington), 10 µg/mL gentamycin (Teknova) and 100 μg/ml primocin (Invivogen).

### Histology

Tissues were fixed in 10% formalin and sectioned at 5 μm^18^. For DAB immunohistochemical (IHC) staining, sections were deparaffinized using standard procedures and antigen retrieval was performed by incubating the samples in 1x citrate buffer pH 6.0 (Diagnostic Biosystems) at 60 °C for 30 min, followed by 15 min cooling at room temperature. Endogenous peroxidase activity was quenched by treating samples with 3% H_2_O_2_ for 10 min at RT. Sections were then blocked in 2.5% normal horse serum for 20 min. Sections were then incubated with primary antibodies diluted in TBS with 0.1–1% BSA (Sigma) overnight at 4 °C. Sections were washed 3 times with TBS + 0.05% Tween 20 (TBST, Sigma) before addition of HRP-conjugated secondary antibody for a 30-min incubation at RT. Sections were washed 3 times with TBST after secondary incubation and DAB substrate was then added until positive signal was observed. Sections were then immediately washed with tap water, counterstained in hematoxylin for 15–30 seconds and washed again with tap water before dehydration and mounting. Isotype controls or secondary antibody only (no primary antibody) controls were used for IHC. For Hematoxylin and Eosin staining, sections were deparaffinized, rinsed in tap water, and stained with Hematoxylin for 3 min. Sections were then washed in tap water and stained with Eosin for 2 min before a final wash in tap water. Safranin O/Fast Green staining was performed as previously described^53,54^. To quantitate cartilage repair, the ICRS II scoring system^35^ was employed by two blinded observers. Toluidine Blue and Alcian Blue staining was performed on deparaffinized sections in accordance with standard laboratory techniques.

### Biomechanical assessment of porcine defect repair

Freshly harvested porcine cartilage tissues 6 months of age were affixed to the sample holder of the Mach-1 Mechanical Tester (Biomomentum) using instant glue (Loctite 4013) and immersed in DMEM/0.9% NaCl (1:1). The Mach-1 configuration uses a spherical indenter tool attached to a highly sensitive multiaxial load cell and automated fine motor controller, allowing for compression of the cartilage by 30% of its thickness, and live recording of resultant forces generated in all x-y-z planes. The indenter tool is then replaced with a needle which penetrates the cartilage at each point until forces comparable to underlying bone are detected, allowing for accurate thickness measurement. Altogether, the forces generated during indentation and thickness measurements are used to calculate the instantaneous modulus, which reflects the elasticity, stiffness, and resistance to compression of the tissue. Each condyle was manually mapped for testing using the Biomomentum mapping software. On average, between 40–50 points were tested on each affected condyle, with no less than 10 points directly in the defect areas, and usually about 1–3 mm apart from each other on the surface. Each control condyle was tested for an average of 15–20 points, which were spaced about 2–5 mm apart. Mapping coordinates were input into the software and indentation analysis to map instantaneous modulus was performed with the 1 mm spherical indentor tool with the following parameters: Z-contact velocity: 0.1000 mm/s; contact criteria: 0.1003 N; scanning grid: 0.2000 mm; indentation amplitude: 0.300 mm; indentation velocity: 0.300 mm/s; relaxation time: 5 s. To map thickness, the indentor tool was replaced with a 26 G ¾ inch hypodermic needle and the mapping executed with the following needle penetration parameters: stage axis: position z; load cell axis: Fz; direction: positive; stage velocity: 0.2000 mm/s; contact criteria: 2.000 N; stage limit: 15 mm; stage repositioning: 2x load resolution; offset: 0. Heat maps were generated with the Mach-1 software provided by Biomomentum.

### Single-cell sequencing using 10X Genomics

Single cell samples were prepared using Single Cell 3^/^ Library & Gel Bead Kit v2 and Chip Kit (10X Genomics) according to the manufacturer’s protocol. Briefly samples were FACS sorted using DAPI to select live cells followed by resuspension in 0.04% BSA-PBS. Nearly 1,200 cells/µl were added to each well of the chip with a target cell recovery estimate of 8,000 cells. Thereafter Gel bead-in Emulsions (GEMs) were generated using GemCode Single-Cell Instrument. GEMs were reverse transcribed, droplets were broken and single stranded cDNA was isolated. cDNAs were cleaned up with DynaBeads and amplified. Finally, cDNAs were ligated with adapters, post-ligation products were amplified, cleaned up with SPRIselect. Purified libraries were submitted to UCLA Technology Center for Genomics & Bioinformatics for quality check and sequencing. The quality and concentration of the purified libraries were evaluated by High Sensitivity D5000 DNA chip (Agilent) and sequencing was performed on NextSeq500.

### 10X sequencing data analysis

Raw sequencing reads were processed using Partek Flow Analysis Software (build version 10.0.21.0210). Briefly, raw reads were checked for their quality, trimmed and reads with an average base quality score per position >30 were considered for alignment. Trimmed reads were aligned to the human genome version hg38-Gencode Genes- release 30 using STAR −2.6.1d with default parameters. Reads with alignment percentage >75% were de-duplicated based on their unique molecular identifiers (UMIs). Reads mapping to the same chromosomal location with duplicate UMIs were removed. Thereafter ‘Knee’ plot was constructed using the cumulative fraction of reads/UMIs for all barcodes. Barcodes below the cut-off defined by the location of the knee were assigned as true cell barcodes and quantified. Further noise filtration was done by removing cells having >3% mitochondrial counts and total read counts >24,000. Genes not expressed in any cell were also removed as an additional clean-up step. Cleaned up reads were normalized using counts per million (CPM) method followed by log transformation generating count matrices for each sample. Samples were batch corrected on the basis of expressed genes and mitochondrial reads percent. Count matrices were used to visualize and explore the samples in further details by generating tSNE plots generated using default parameters in Partek. K-means clustering was computed for identifying groups of cells with similar expression profile using Euclidean distance metric based on the most appropriate cluster count. A maximum of 1000 iterations were allowed and the top marker features for each cluster was determined.

Gene ontology enrichment analysis for the differentially expressed genes was performed using DAVID Gene Functional Classification Tool (http://david.abcc.ncifcrf.gov; version 6.8). Dot plots and Violin plots were generated in R (v4.0.3) using ggplot2 (v3.3.3) package. Two-way Venn diagrams were generated using BioVenn^55^. Hypergeometric p values were calculated assuming 25,000 human genes.

Cell trajectories were constructed using Monocle3 package in R according to Trapnell lab guidelines^48^ (https://github.com/cole-trapnell-lab/monocle3). Count matrices, cell metadata and gene metadata were used to create Monocle3 object. A subset of genes that exhibit high cell-to-cell variation in the dataset was determined by directly modeling the mean-variance relationship inherent in the data using Seurat^56^. Pre-processing was done by calculating significant PCs (principal components) ensuring usage of enough PCs to capture most of the variation in gene expression across all the cells in the data set. Variable genes obtained from Seurat were used to pre-process the data. The dimensionality of the data was thereafter reduced using uniform manifold approximation and projection (UMAP). Cells were plotted onto the UMAP space for visualization of their distribution, identification of cell types and community detection to group cells into clusters. Next the principal graph was learned within each cluster, trajectory was constructed and the cells were ordered to measure their progress in pseudotime.

### Methylcellulose culture method of porcine BMSCs and chondrocytes

To assess the clonality and capacity of pig BMSCs to undergo chondrogenesis in vitro, porcine bone marrow stromal cells (pBMSCs) or articular chondrocytes were isolated from the distal femoral epiphysis or articular surface of the condyles, respectively, of 3–4-month-old Yucatan minipigs (S & S Farms). Tissues were digested as described above. Methylcellulose-based media (StemCell) was resuspended with DMEM/F12 (Corning) + 10% FBS + 1% P/S/A and either 10 ng/ml FGF-2, 10 ng/ml BMP-2, 10 ng/ml TGFβ-1, or all three growth factors, to make a 1% methylcellulose-based media. Either P1 BMSCs or P0 chondrocytes were seeded in 6-well ultra-low attachment plates (Corning) at a low density (300 cells / ml) and cultured for 3–4 weeks. 120–160 μL of liquid DMEM/F12 with respective growth factors was applied to the surface of the wells to ensure moisture and nutrients remained available to the cells biweekly. For the Transwell-methylcellulose co-culture, hES-derived chondrocytes on membranes were placed in a 1 μm-pore Transwell insert (Falcon) with DMEM/F12 + 10% FBS + 1% P/S/A. P1 MSCs or P0 chondrocytes were seeded in the same media with methylcellulose, and cultured in 24-well ultra-low attachment plates (Corning) at a low density (300 cells / ml) for 3–4 weeks. 40–60 μL of media was added biweekly to the methylcellulose as maintenance. Clonogenicity was calculated by manual counting of clones larger than ~40 μm in diameter or greater than 5 cell divisions. All images of clones in methylcellulose were taken on an Echo Revolve Inverted microscope.

### ELISA detection of proteins secreted by hESDC-M

Either 1 × 10^6^ human bone marrow stromal cells (hBMSCs; *n* = 7 different donors) or whole hES-derived chondrocytes on membranes (*n* = 6–9 batches) were lysed with 500 μL of 2X Lysis buffer (Ray Biotech) supplemented with a phosphatase/protease inhibitor (Thermo-Fisher). Lysates were then centrifuged to remove cellular debris, and a Bicinchoninic acid (BCA) protein assay (Thermo-Fisher) was performed to quantify total lysed protein. ELISAs for FGF-2 (Ray Biotech), BMP-2 (R & D Systems), and TGF-β1 (R & D Systems) were performed according to the manufacturer’s protocols.

### Antibody list

Please see Table 2 for a list of antibodies and dilutions used.

**Table 2:** Antibodies used in this study.

Antibody	Vendor	Catalog Number	Dilution
CD326-PerCP-Cy5.5	BD Biosciences	347199	10 uL/10^6^ cells
CD309-PE	R&D Systems	FAB357P	10 uL/10^6^ cells
Collagen II	Abcam	ab185430	1:100–1:250 (IHC)
PRG4	Abcam	ab28484	1:250 (IHC)
SOX9	Abcam	ab26414	1:200 (IHC)
Collagen X	Abcam	ab58632	1:250–1:1000 (IHC)
Collagen I	Abcam	ab34710	1:250 (IHC)
Ku80	Abcam	ab79391	1:250 (IHC)
CD3	Protein Tech	17617-1-AP	1:500 (IHC)
CD68	Bioss	BS-1432R	1:50 (IHC)
Myeloperoxidase	Invitrogen	PA5-16672	1:50 (IHC)
Anti-Mouse IgG ImmPRESS	Vector	MP-7422	Pre-diluted
Anti-Rabbit IgG ImmPRESS	Vector	MP-7401	Pre-diluted
Mouse IgG Isotype Control	abCAM	Ab170191	1:50–1:1000 (IHC)
Rabbit IgG Isotype Control	Invitrogen	02-6102	1:50–1:1000 (IHC)

### Reporting summary

Further information on research design is available in the Nature Research Reporting Summary linked to this article.

## Supplementary information

Supplementary Information

Reporting Summary

## Data Availability

All scRNA-sequencing data are deposited in GEO under accession GSE142045.
