# Supplementary material for: Long-term repair of porcine articular cartilage using cryopreservable, clinically compatible human embryonic stem cell-derived chondrocytes
Source: NPJ Regen Med. 2021 Nov 23;6:77. doi: 10.1038/s41536-021-00187-3 (PMC8611001; doi:10.1038/s41536-021-00187-3)
Supplement: Supplementary file 1 — Supplementary Information [file 41536_2021_187_MOESM1_ESM.pdf]

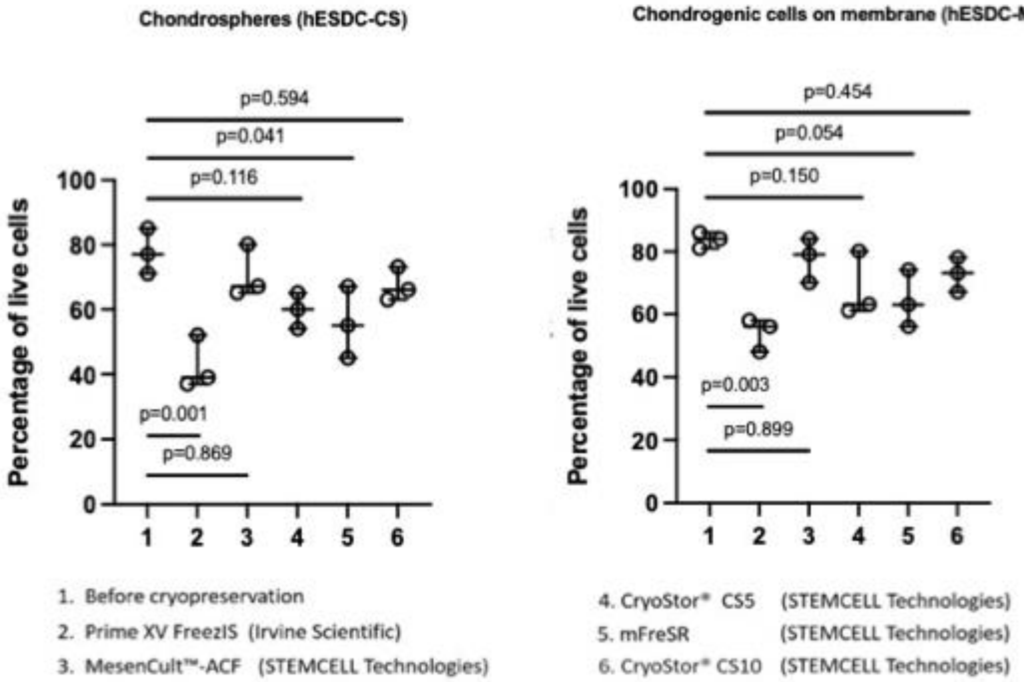

Supplementary Figure 1: Optimization of cell cryopreservation. Cell viability was assessed using Live/Dead assay prior to viably freezing and within 4 hours after thawing. Mesencult™ ACF provided the best viability post-thaw and was used for all subsequent preparations. p values were calculated via one-way ANOVA followed by Tukey's test; data are presented as mean $\pm$ SD of 3 experimental replicates.

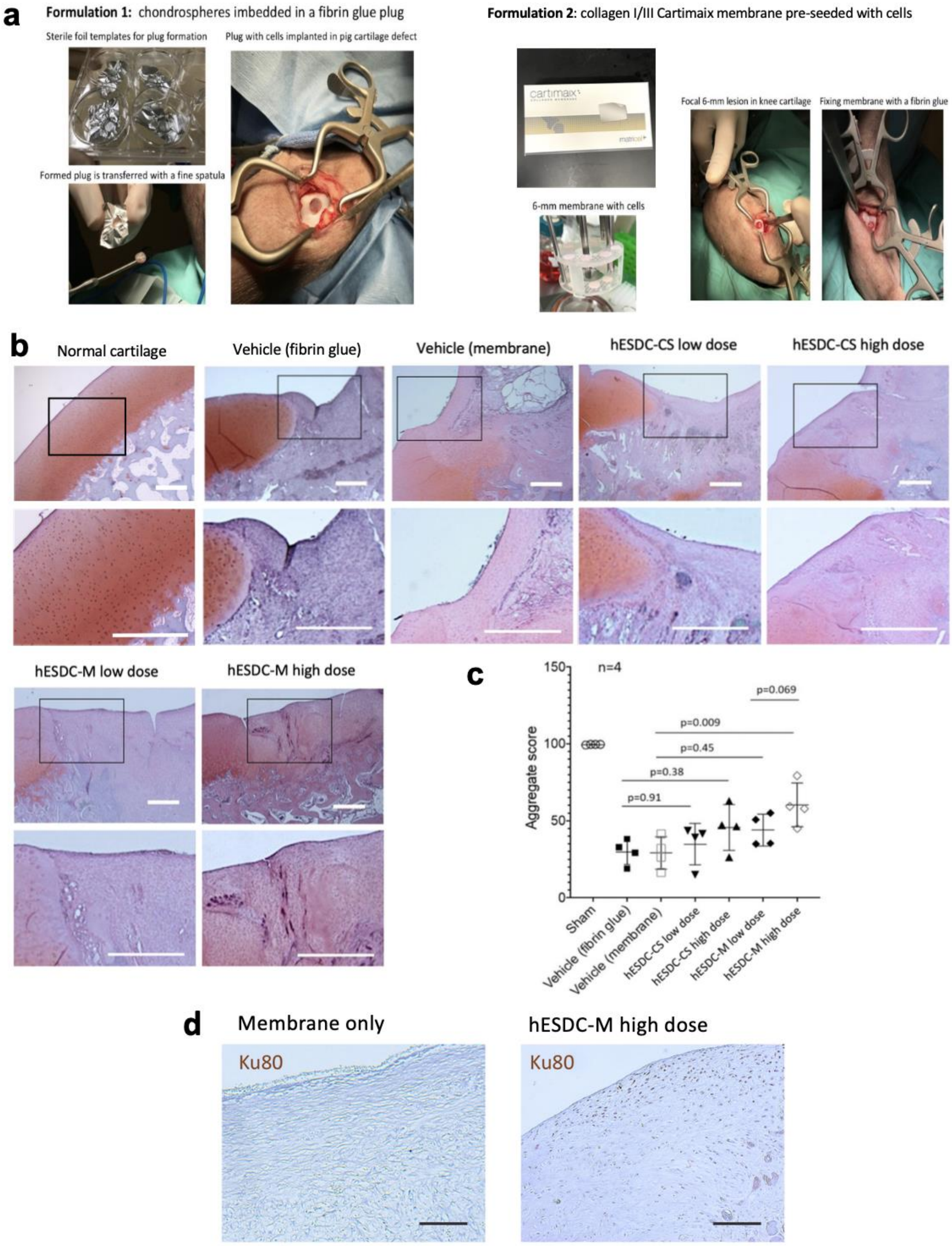
a
Formulation 1: chondrospheres imbedded in a fibrin glue plug
Sterile foil templates for plug formation
Plug with cells implanted in pig cartilage defect
Formed plug is transferred with a fine spatula
Formulation 2: collagen I/III Cartimaix membrane pre-seeded with cells
6-mm membrane with cells
Focal 6-mm lesion in knee cartilage
Fixing membrane with a fibrin glue
b
Normal cartilage
Vehicle (fibrin glue)
Vehicle (membrane)
hESDC-CS low dose
hESDC-CS high dose
hESDC-M low dose
hESDC-M high dose
c
n=4
Aggregate score
150
100
50
0
p=0.069
p=0.009
p=0.45
p=0.38
p=0.91
Sham
Vehicle (fibrin glue)
Vehicle (membrane)
hESDC-CS low dose
hESDC-CS high dose
hESDC-M low dose
hESDC-M high dose
d
Membrane only
hESDC-M high dose
Ku80
Ku80

Supplementary Figure 2: Overview of surgical procedure and assessment of short-term cartilage repair at 1 month. (a) Full-thickness defects were created in Yucatan minipig knees and transplanted with low and high doses of either chondrospheres (CS) or hESDC-M. (b) Assessment of proteoglycan content via Safranin O staining of healthy cartilage and groups one month after implantation; images show the junction of intact articular cartilage with the defect area, with boxes denoting the regions shown at higher magnification. (c) ICRS II aggregate scoring of defects calculated via 14 parameters of the ICRS II Cartilage Repair Scoring System demonstrated the high dose, membrane embedded formulation of hES-derived chondrocytes provided superior short-term repair. (d) Representative images of $Ku80^+$ human cells in the membrane only or hESDC-M after 1 month. n=4 defects per condition, 2 defects per knee. Scale bars = 100 µm; data presented as mean ± SD. p values were calculated via one-way ANOVA followed by Tukey's test.

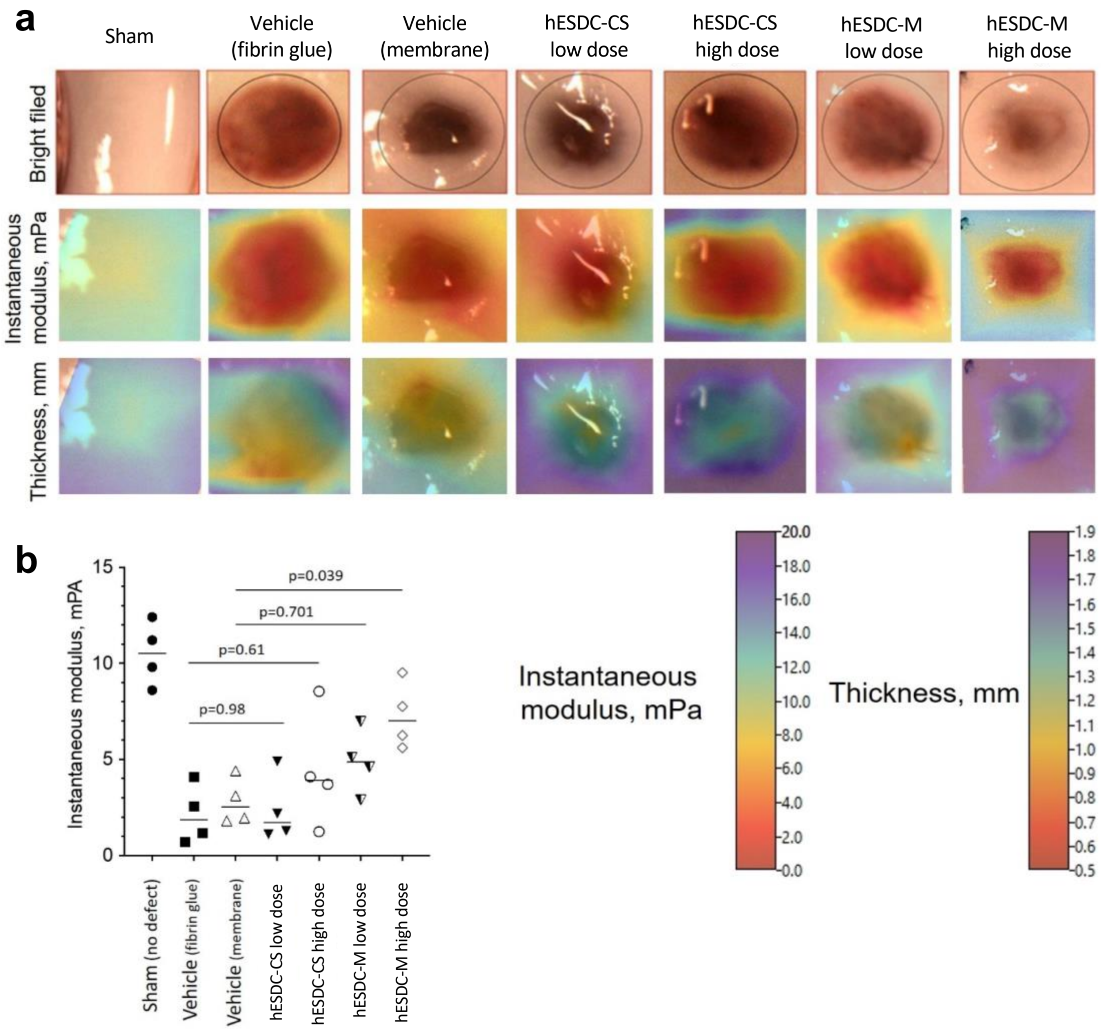
a
Sham
Vehicle (fibrin glue)
Vehicle (membrane)
hESDC-CS low dose
hESDC-CS high dose
hESDC-M low dose
hESDC-M high dose
Bright filed
Instantaneous modulus, mPa
Thickness, mm
b
Instantaneous modulus, mPA
15
10
5
0
p=0.039
p=0.701
p=0.61
p=0.98
Sham (no defect)
Vehicle (fibrin glue)
Vehicle (membrane)
hESDC-CS low dose
hESDC-CS high dose
hESDC-M low dose
hESDC-M high dose
Instantaneous modulus, mPa
20.0
18.0
16.0
14.0
12.0
10.0
8.0
6.0
4.0
2.0
0.0
Thickness, mm
1.9
1.8
1.7
1.6
1.5
1.4
1.3
1.2
1.1
1.0
0.9
0.8
0.7
0.6
0.5

Supplementary Figure 3: Biomechanical properties of repaired cartilage defects. (a) Analysis of biomechanical properties was carried out using Mach-1 scanning indenter (Biomomentum, Canada). At least 10 points were analyzed within each defect. Heat maps representing instantaneous modulus and thickness are shown for each group. Color mapping is artificial to illustrate the differences between specimens; scales for each measurement are shown below. (b) Quantitative assessment of instantaneous modulus within the healing defect area 1 month after transplantation of 2 doses of either hESC-derived chondrospheres (hESDC-CS) or collagen I/III membrane embedded hESDC-M. The best repair was observed in the high dose hESDC-M group. Data presented as mean ± SD of aggregate values for 4 defects per each group; p values were calculated via one-way ANOVA followed by Tukey's test.

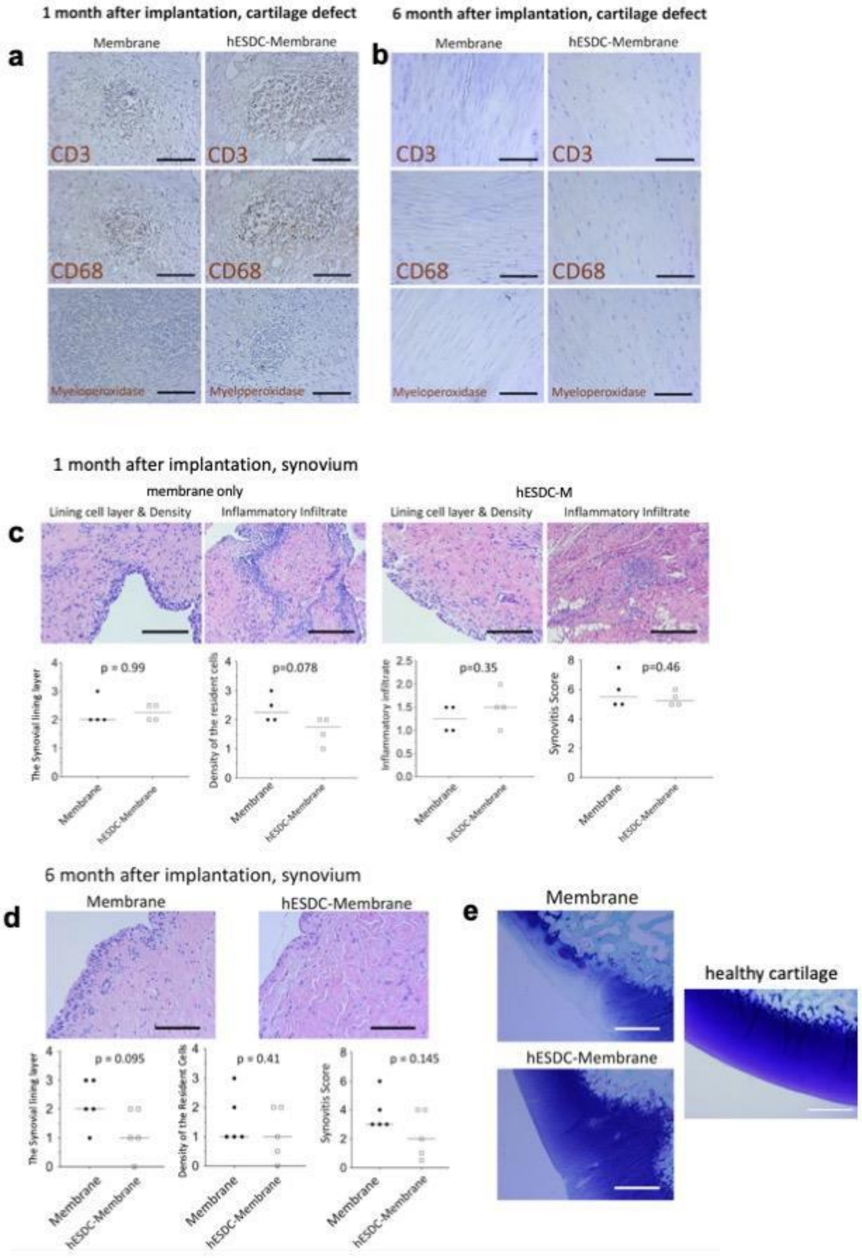
1 month after implantation, cartilage defect
6 month after implantation, cartilage defect
Membrane
hESDC-Membrane
a
b
CD3
CD68
Myeloperoxidase
1 month after implantation, synovium
membrane only
hESDC-M
Lining cell layer & Density
Inflammatory Infiltrate
c
p = 0.99
p=0.078
p=0.35
p=0.46
The Synovial lining layer
Density of the resident cells
Inflammatory infiltrate
Synovitis Score
6 month after implantation, synovium
d
e
p = 0.095
p = 0.41
p = 0.145
Density of the Resident Cells
healthy cartilage

Supplementary Figure 4: Immunological response to hESDC-M. (a) Representative images of immunohistochemical staining for immune cell markers in the cartilage defect at 1 month post implantation of the membrane only (left column) or the hESDC-M (right column). (b) Representative images of immunohistochemical staining for immune cell markers in the cartilage defect at 6 months post implantation of the membrane only (left column) or the hESDC-M (right column). (c) Representative Hematoxylin and Eosin (H&E) stains and quantification of synovial characteristics 1 month after implantation using previously described methods[29] (n=4 biological replicates for membrane only and hESDC-M group (d) Representative H&E stain and corresponding quantification of synovium 6 months after implantation; left image is the membrane only, right image is the defect with hESDC-M; (n=5 biological replicates for membrane only group, n=5 for hESDC-M group) (e) Representative Toluidine Blue stain of healthy articular cartilage and cartilage with the defect area 6 months after implantation; top image is the membrane only, bottom image is the defect with hESDC-M, and right is the healthy cartilage. All scale bars = 100 μm, p values calculated with an unpaired t-test and data is presented as mean ± SD.

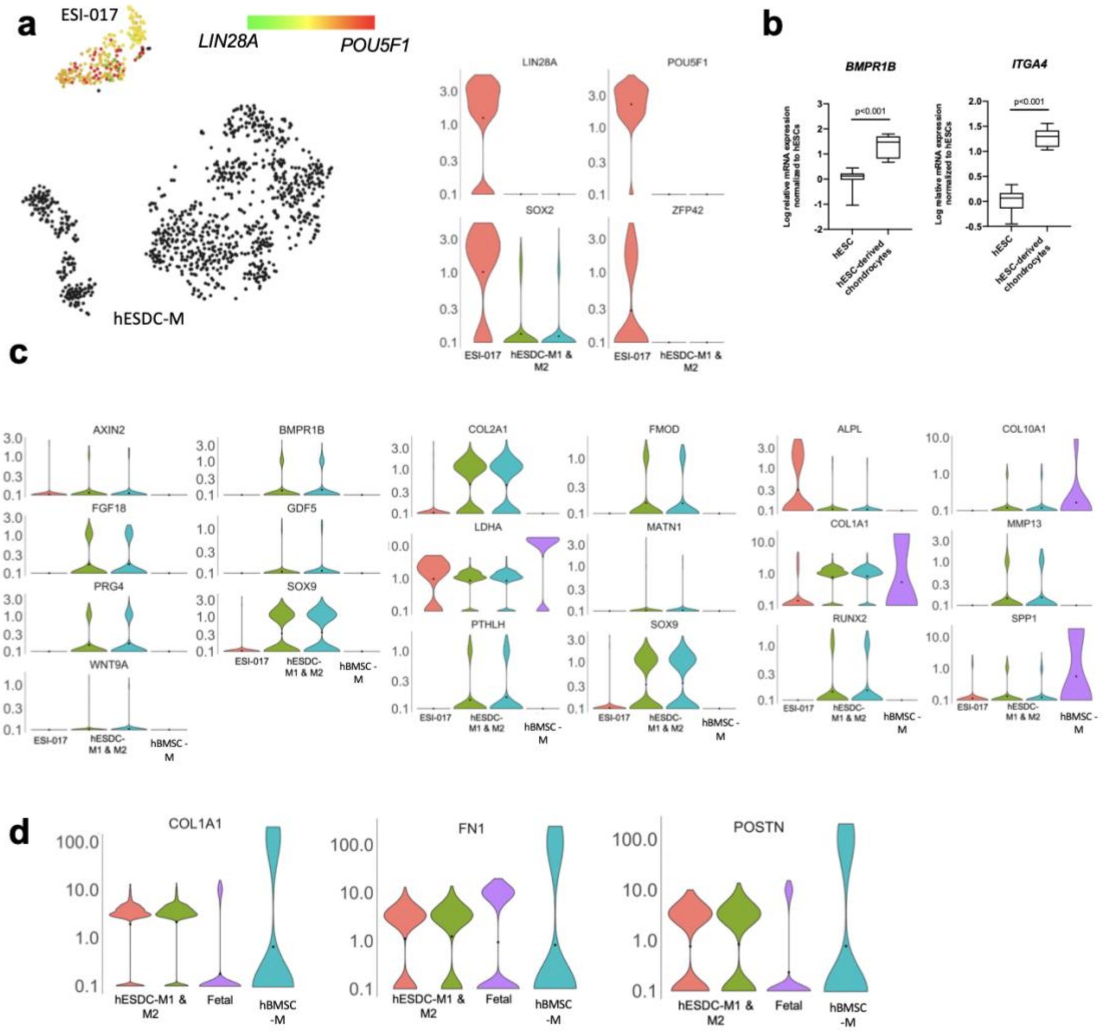

Supplementary Figure 5. hESDC-M are not contaminated by hESCs and represent more immature chondrogenic cells than hBMSC-M. (a) t-SNE and violin plots depicting expression of indicated genes at single cell resolution. (b) qPCR of superficial gene expression (n=7 independent batches; p values were calculated with an unpaired t-test; data presented as box and whisker plots. (c) Violin plots for gene expression of selected chondrogenic genes in hESCs, hESDC-M and human bone marrow stromal cells cultured on membranes (hBMSCs-M). (d) Violin plots for gene expression of selected stromal genes.

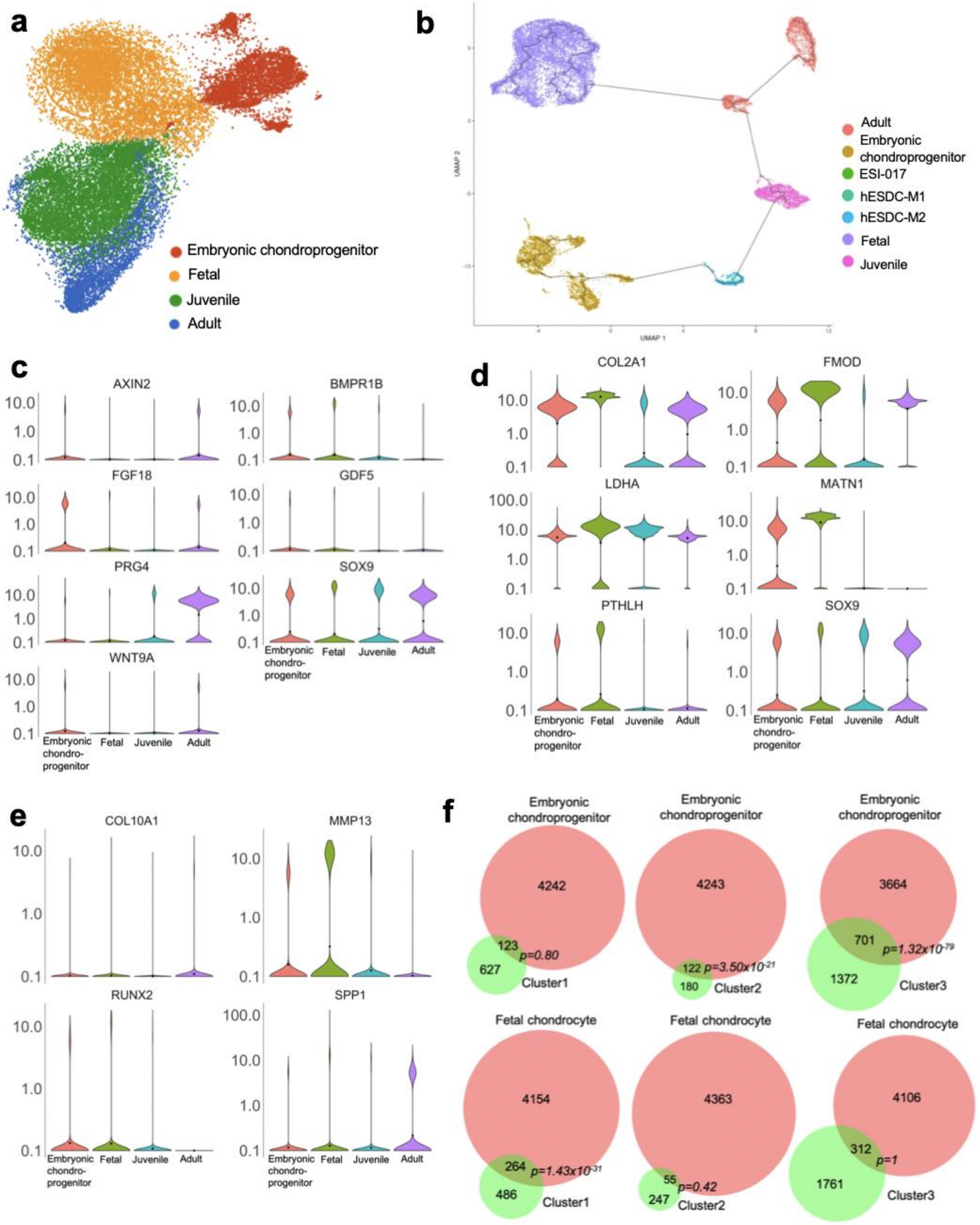
a
Embryonic chondroprogenitor
Fetal
Juvenile
Adult
b
Adult
Embryonic chondroprogenitor
ESI-017
hESDC-M1
hESDC-M2
Fetal
Juvenile
c
AXIN2
BMPR1B
FGF18
GDF5
PRG4
SOX9
WNT9A
Embryonic chondro-progenitor
Fetal
Juvenile
Adult
d
COL2A1
FMOD
LDHA
MATN1
PTHLH
SOX9
e
COL10A1
MMP13
RUNX2
SPP1
f
Embryonic chondroprogenitor
4242
123
p=0.80
627
Cluster1
4243
122
p=3.50x10-21
180
Cluster2
3664
701
p=1.32x10-79
1372
Cluster3
Fetal chondrocyte
4154
264
p=1.43x10-31
486
Cluster1
4363
55
p=0.42
247
Cluster2
4106
312
p=1
1761
Cluster3

Supplementary Figure 6. Definition of chondrogenic ontogeny at the single cell level. (a) t-SNE plot of single cell sequencing data generated at 4 stages of human chondrocyte ontogeny. (b) Cell trajectory analysis of Col2^{+} cells from human *in vivo* ontogeny and cultured hESDC-M and ESI-017 cells constructed using Monocle3[48]. Violin plots for gene expression of selected (c) superficial, (d) transitional and (e) deep zone genes. (f) Gene sets were created by intersecting two previously published data sets[17,18] generated with bulk RNA-seq of embryonic chondroprogenitors and fetal chondrocytes. Genes enriched in "pre-chondrocytes" vs. "resting chondrocytes" were overlapped with genes enriched in "embryonic 5-6 WPC (weeks post conception)" and "17 WPC" and vice versa to create lists of common genes enriched in embryonic chondroprogenitors and fetal chondrocytes. Venn diagrams demonstrating overlap of biomarker genes strongly enriched and representative of the indicated cluster of hESDC-M (see Figure 2h) analyzed by scRNA-Seq with gene lists generated by bulk RNA-Seq.

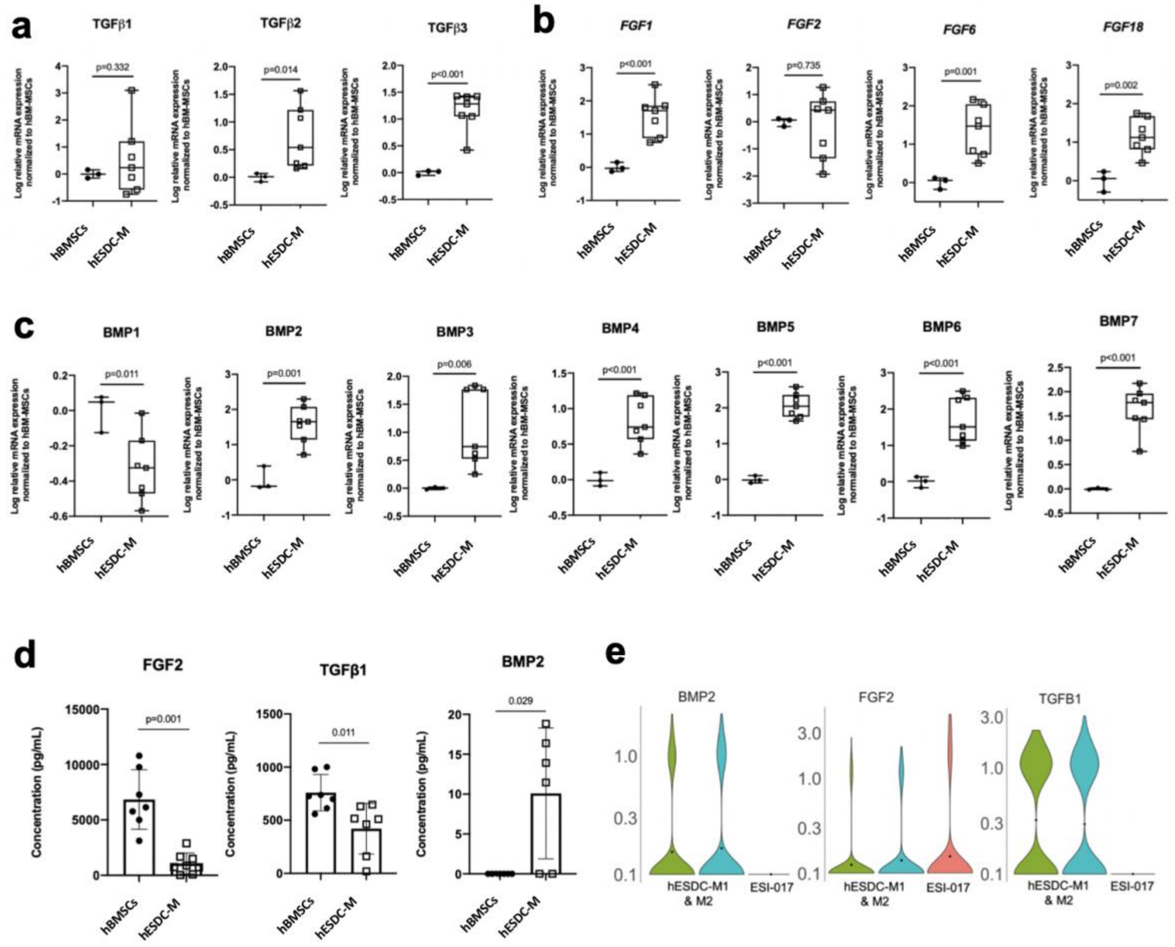

Supplementary Figure 7: Assessing levels of chondroinductive paracrine factors of hESDC-M and hBMSCs. qPCR for various (a) TGF-**β**, (b) FGF, and (c) BMP family members in P1 hBMSCs (n=3 biological replicates of 27-29 yo) and hESDC-M (n=7 batches). (d) ELISA analyses of representative growth factors (BMP-2, TGF-**β**1, & FGF-2) secreted by P1-3 hBMSCs (n=7 biological replicates aged 19-64 yo) and hESDC-M (n=9 batches for FGF2, n=7 batches for TGF-**β**1 and n=6 batches for BMP-2). p values calculated with unpaired t-test; error bars represented as mean ± SD. (e) Violin plots depicting gene expression in ESI-017 cells and 2 replicates of hESDC-M analyzed by scRNA-Seq.

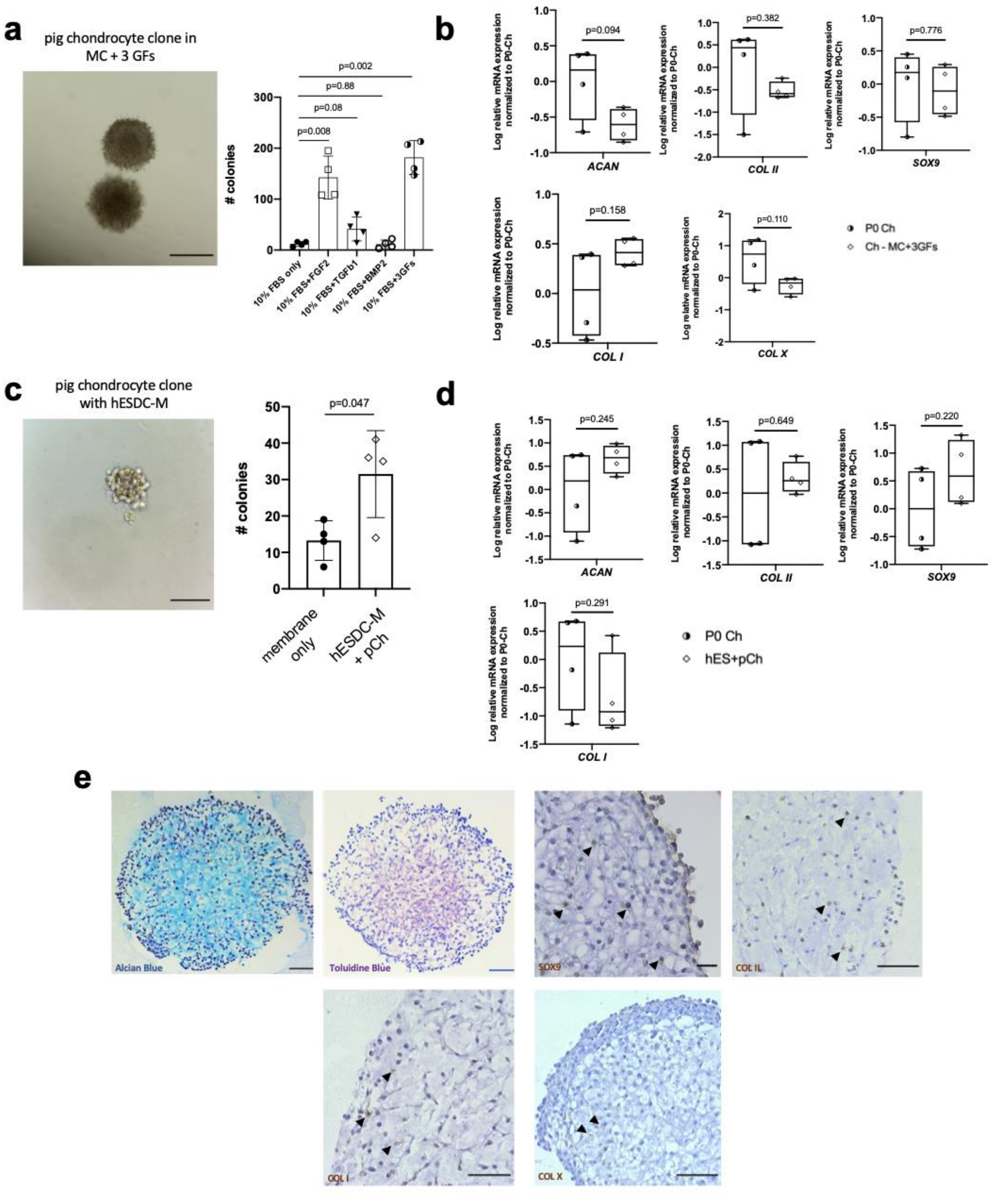
a
pig chondrocyte clone in
MC + 3 GFs
# colonies
p=0.002
p=0.88
p=0.08
p=0.008
10% FBS only
10% FBS+FGF2
10% FBS+TGFb1
10% FBS+BMP2
10% FBS+3GFs
b
Log relative mRNA expression normalized to P0-Ch
p=0.094
ACAN
p=0.382
COL II
p=0.776
SOX9
p=0.158
COL I
p=0.110
COL X
P0 Ch
Ch - MC+3GFs
c
pig chondrocyte clone
with hESDC-M
# colonies
p=0.047
membrane only
hESDC-M + pCh
d
Log relative mRNA expression normalized to P0-Ch
p=0.245
ACAN
p=0.649
COL II
p=0.220
SOX9
p=0.291
COL I
P0 Ch
hES+pCh
e
Alcian Blue
Toluidine Blue
SOX9
COL II
COL I
COL X

Supplementary Figure 8: Endogenous articular chondrocytes maintain their chondrogenic profile in the presence of hESDC-M. (a) Representative image of two pig chondrocyte (Ch) colonies (scale bar = 500 µm) & clonogenicity of pig chondrocytes (n=4 biological replicates) cultured in methylcellulose (MC) for 4 weeks. (b) qPCR of chondrogenic genes (n=4 biological replicates). (c) Representative image of a pig Ch colony in MC after 4 weeks of culture with hESDC-M in a Transwell. Clonogenicity of pig Ch in MC with either an empty membrane or hESDC-M in a Transwell after 4 weeks (n=4 biological replicates per group). Scale bar = 100 µm. (d) qPCR of chondrogenic genes in pig Ch grown in Transwell culture with hESDC-M (n=4 biological replicates). (e) Alcian Blue and Toluidine Blue staining (left, middle left) and immunohistochemical staining of various chondrogenic markers of pig chondrocytes grown in MC with 3 GFs after 4 weeks. Scale bar = 100 µm; all p values calculated with unpaired t-test; error bars represented as mean ± SD.

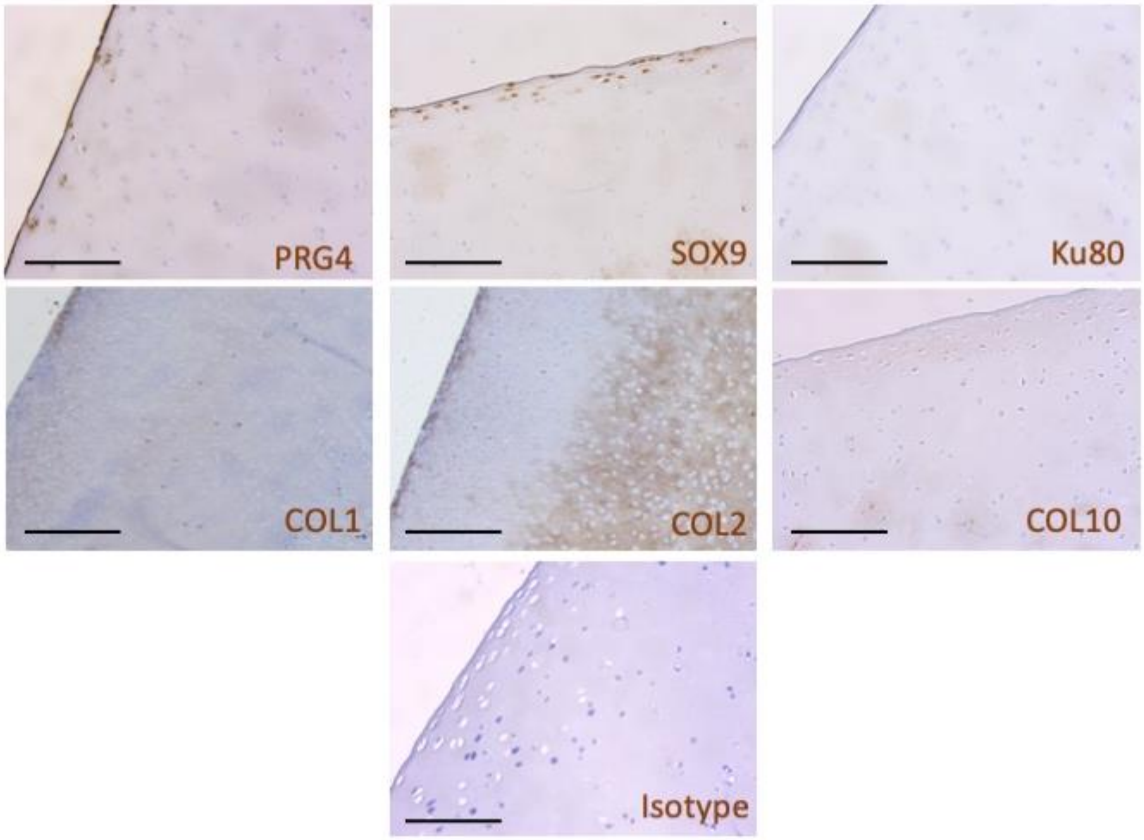

Supplementary Figure 9: Immunohistochemical characterization of healthy porcine cartilage. Representative images of immunohistochemical staining of healthy porcine articular cartilage for human-specific antigen Ku80 and cartilage zonal markers; scale bar = 200 μm.
