# Supplementary material for: Long-term repair of porcine articular cartilage using cryopreservable, clinically compatible human embryonic stem cell-derived chondrocytes
Source: NPJ Regen Med. 2021 Nov 23;6:77. doi: 10.1038/s41536-021-00187-3 (PMC8611001; doi:10.1038/s41536-021-00187-3)
Supplement: Supplementary file 2 — Reporting Summary [file 41536_2021_187_MOESM2_ESM.pdf]

nature portfolio

Corresponding author(s): Denis Evseenko

Last updated by author(s): 10-07-21

Reporting Summary

Nature Portfolio wishes to improve the reproducibility of the work that we publish. This form provides structure for consistency and transparency in reporting. For further information on Nature Portfolio policies, see our Editorial Policies and the Editorial Policy Checklist.

Statistics

For all statistical analyses, confirm that the following items are present in the figure legend, table legend, main text, or Methods section.

n/a	Confirmed	
☐	☒	The exact sample size (*n*) for each experimental group/condition, given as a discrete number and unit of measurement
☐	☒	A statement on whether measurements were taken from distinct samples or whether the same sample was measured repeatedly
☐	☒	The statistical test(s) used AND whether they are one- or two-sided<br>*Only common tests should be described solely by name; describe more complex techniques in the Methods section.*
☐	☒	A description of all covariates tested
☐	☒	A description of any assumptions or corrections, such as tests of normality and adjustment for multiple comparisons
☐	☒	A full description of the statistical parameters including central tendency (e.g. means) or other basic estimates (e.g. regression coefficient) AND variation (e.g. standard deviation) or associated estimates of uncertainty (e.g. confidence intervals)
☐	☒	For null hypothesis testing, the test statistic (e.g. *F*, *t*, *r*) with confidence intervals, effect sizes, degrees of freedom and *P* value noted<br>*Give P values as exact values whenever suitable.*
☒	☐	For Bayesian analysis, information on the choice of priors and Markov chain Monte Carlo settings
☒	☐	For hierarchical and complex designs, identification of the appropriate level for tests and full reporting of outcomes
☒	☐	Estimates of effect sizes (e.g. Cohen's *d*, Pearson's *r*), indicating how they were calculated

Our web collection on statistics for biologists contains articles on many of the points above.

Software and code

Policy information about availability of computer code

Data collection	No software/code was used.
Data analysis	No software/code was used.

For manuscripts utilizing custom algorithms or software that are central to the research but not yet described in published literature, software must be made available to editors and reviewers. We strongly encourage code deposition in a community repository (e.g. GitHub). See the Nature Portfolio guidelines for submitting code & software for further information.

Data

Policy information about availability of data

All manuscripts must include a data availability statement. This statement should provide the following information, where applicable:

- Accession codes, unique identifiers, or web links for publicly available datasets
- A description of any restrictions on data availability
- For clinical datasets or third party data, please ensure that the statement adheres to our policy

All scRNA-sequencing data are deposited in GEO under accession GSE142045.

Field-specific reporting

Please select the one below that is the best fit for your research. If you are not sure, read the appropriate sections before making your selection.

☒ Life sciences ☐ Behavioural & social sciences ☐ Ecological, evolutionary & environmental sciences

For a reference copy of the document with all sections, see nature.com/documents/nr-reporting-summary-flat.pdf

Life sciences study design

All studies must disclose on these points even when the disclosure is negative.

Sample size	Please see the 'general methods' subsection, paragraph 1, and 'Large animal model of articular cartilage repair' subsection.
Data exclusions	All samples were included.
Replication	For all experiments, biological replicates were employed to generate data. For in vitro experiments expected to yield large differences, standard practice of using 3 replicates was followed.
Randomization	No randomization was used.
Blinding	N/A

Reporting for specific materials, systems and methods

We require information from authors about some types of materials, experimental systems and methods used in many studies. Here, indicate whether each material, system or method listed is relevant to your study. If you are not sure if a list item applies to your research, read the appropriate section before selecting a response.

Materials & experimental systems

n/a	Involved in the study	
☐	☒	Antibodies
☐	☒	Eukaryotic cell lines
☒	☐	Palaeontology and archaeology
☐	☒	Animals and other organisms
☒	☐	Human research participants
☒	☐	Clinical data
☒	☐	Dual use research of concern

Methods

n/a	Involved in the study	
☒	☐	ChIP-seq
☒	☐	Flow cytometry
☒	☐	MRI-based neuroimaging

Antibodies

Antibodies used	Catalog numbers provided in Methods 'Table 2" subsection.
Validation	Validation is per manufacturer's commercial guidelines and can be found online.

Eukaryotic cell lines

Policy information about cell lines

Cell line source(s)	Embryonic Stem Cell
Authentication	Purchased GMP-grade, authenticated
Mycoplasma contamination	Please see methods 'Culture of pluripotent stem cells and generation of skeletal progenitors' subsection.
Commonly misidentified lines (See ICLAC register)	N/A

Animals and other organisms

Policy information about studies involving animals; ARRIVE guidelines recommended for reporting animal research

Laboratory animals	Please see methods 'Large animal model of articular cartilage repair' subsection.

Wild animals	N/A
Field-collected samples	N/A
Ethics oversight	Please see methods 'Large animal model of articular cartilage repair' subsection.

Note that full information on the approval of the study protocol must also be provided in the manuscript.
